# Modeling the Structure and Interactions of Intrinsically Disordered Peptides with Multiple Replica, Metadynamics-Based Sampling Methods and Force-Field Combinations

**DOI:** 10.1021/acs.jctc.1c00889

**Published:** 2022-02-17

**Authors:** Lunna Li, Tommaso Casalini, Paolo Arosio, Matteo Salvalaglio

**Affiliations:** † Thomas Young Centre and Department of Chemical Engineering, University College London, London WC1E 7JE, U.K.; ‡ Department of Chemistry and Applied Biosciences, ETH Zurich, Zurich 8093, Switzerland

## Abstract

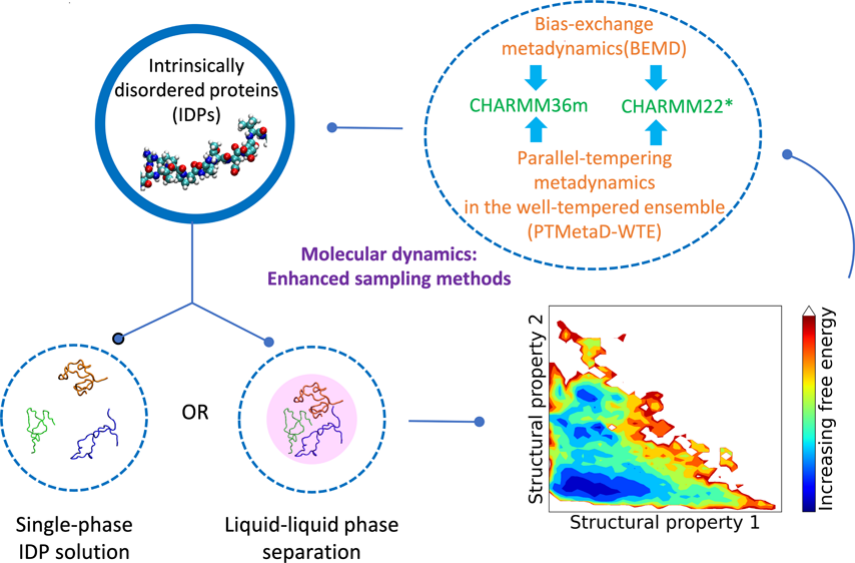

Intrinsically disordered proteins play a key role in many biological processes, including the formation of biomolecular condensates within cells. A detailed characterization of their configurational ensemble and structure–function paradigm is crucial for understanding their biological activity and for exploiting them as building blocks in material sciences. In this work, we incorporate bias-exchange metadynamics and parallel-tempering well-tempered metadynamics with CHARMM36m and CHARMM22* to explore the structural and thermodynamic characteristics of a short archetypal disordered sequence derived from a DEAD-box protein. The conformational landscapes emerging from our simulations are largely congruent across methods and force fields. Nevertheless, differences in fine details emerge from varying combinations of force-fields and sampling methods. For this protein, our analysis identifies features that help to explain the low propensity of this sequence to undergo self-association in vitro, which are common to all force-field/sampling method combinations. Overall, our work demonstrates the importance of using multiple force-field and sampling method combinations for accurate structural and thermodynamic information in the study of disordered proteins.

## Introduction

Intrinsically disordered proteins (IDPs) are abundantly present in nature and play important roles in diverse biological functions, including cellular signaling and regulation of gene expression.^1−8^ A class of IDPs, defined as low complexity domains (LCDs), have been recently discovered in association with the dynamic formation of open compartments in cells.^4−8^ These compartments, which are often defined as condensates, are associated with liquid–liquid phase separation (LLPS) of proteins and nucleic acids and could underlie important functions and dysfunctions in biology. In addition to their role in biology, these LCDs represent promising building blocks for the design of synthetic organelles capable to encode novel biochemical functionalities in a controllable and programmable manner, either alone or conjugated to soluble globular domains.^9−15^

IDPs can be characterized by the lack of stable well-defined native structures of folded proteins^16^ and amino-acid compositions biased towards a high fraction of charged and polar residues, secondary structure disrupters such as proline and glycine, and a low amount of bulky hydrophobic amino acids.^1,15−20^ It is believed that the multivalent attractive interactions between side chains may give rise to favorable energetic gain that is responsible for counteracting the entropic loss during LLPS,^15,21,22^ and the phase behaviors of IDPs can be specifically encoded in their protein sequences.^22−32^ Nevertheless, our understanding of IDPs and their key role in mediating phase separations of multicomponent structures into coacervate assemblies is still largely limited. In the literature, mean-field theories such as Flory–Huggins theory and a recently emerging sticker-and-spacer model provide highly generalizable frameworks for describing the thermodynamics of the phase behavior of associative polymers in solution,^33,34^ with emerging applications to complex biological systems both experimentally and computationally.^34−37^ The thermodynamic driving force towards phase separation is likely to be inherently determined by the conformational free energy (FE) landscape of individual IDPs (intramolecular factor) and their molecular interactions with other sequences in solution including their own replicates (intermolecular factor). Thus, uncovering protein dynamics and their structure–function relationship can be pivotal for establishing versatile and sensitive protocols to design, program, and predict the bottom-up assembly of multifunctional bio-inspired protein-based materials based on IDPs.

Molecular dynamics (MD) simulations can in principle offer the opportunity of resolving structural, kinetic, and thermodynamic characteristics of biological systems such as IDPs and IDP-rich bodies at the length and time scales that are not easily accessible experimentally.^23,38−52^ Recently, many protein folding problems were successfully solved by the advancement of the deep learning method AlphaFold.^53^ However, IDPs generally do not adopt a stably folded structure, featuring FE landscapes containing a number of minima for different competing low-energy structures.^16,17^ Thus, exploring the conformations of IDPs still largely rely on MD simulations.^16^ MD simulations complement state-of-the-art experimental methods such as NMR spectroscopy,^54,55^ single-molecule Förster resonance energy transfer (sm-FRET),^56,57^ and small-angle X-ray and neutron scattering (SAXS and SANS),^58,59^ which may exhibit challenges in measuring molecular motions at atomic resolution and conformational heterogeneity associated with structural disorder.^38,42,43,50^ Nevertheless, conformational sampling in classical MD simulations can be restrained to local minima of FE surfaces (FESs), and accessing the full and complex landscape of disordered proteins can be nontrivial with standard unbiased simulations. To mitigate these problems, various enhanced sampling schemes have been developed to help probe the regions of the configurational space that could be rarely explored otherwise. Unbiased enhanced samplings are largely based upon the concept of parallel tempering (PT).^51,52^ In the temperature PT/replica-exchange algorithm, multiple replicas are simulated at different temperatures, in which conformations are exchanged at regular intervals, based on the Metropolis acceptance criterion. The stochastic nature of the method ensures generation of the Boltzmann-weighted ensemble from which thermodynamic averages can be straightforwardly obtained. However, temperature differences between neighboring replicas must be moderate in order to yield practically large acceptance rates. For very large biomolecular systems simulated in explicit solvent, the number of replicas needed increases as *O*(*f*^1/2^) for a system with *f* degrees of freedom, so reaching high-temperature ensembles requires challenging computational costs.^52,60^ In Replica Exchange with Solute Tempering (REST2),^38,41,46,61,62^ such issue can be overcome by scaling the protein intramolecular potential energy, in which case the acceptance probability depends only on the differences of intramolecular solute energy and intermolecular solute water energy. Recently, Replica exchange with hybrid tempering (REHT)^42^ was developed to enhance exploring complex FE landscape with large FE barriers in the replica-exchange framework. At the same time, metadynamics-based techniques are powerful tools to accelerate the exploration of rare events such as protein folding and conformational transitions.^23,39,40,63−72^ These methods promote the exploration of FE minima by iteratively building a history-dependent bias potential as a sum of Gaussians defined as a function of a chosen set of collective variables (CVs). The Boltzmann-weighted configuration ensemble can then be obtained via appropriate reweighting techniques.^73−75^ Among the many versions of metadynamics-based methods, well-tempered metadynamics (MetaD-WTE) allows the Gaussian height to be decreased with time so that the bias potential smoothly converges to the exact FE surface in the long-time limit,^68,69^ while Metadynamics with Adaptive Gaussians can adapt the Gaussian width on the fly to the local features of FESs in order to improve sampling efficiency.^63^

Here, we critically compare the application combinations of two metadynamics-based, multiple-replica sampling methods and two protein force fields to develop a systematic understanding of the conformational ensemble of an IDP. The methods chosen are bias-exchange metadynamics (BEMD)^66,67^ and PT well-tempered metadynamics (PTMetaD-WTE),^39,47,68−70^ which merge the advantages of the PT method and metadynamics-based techniques. PTMetaD-WTE can significantly reduce the number of replicas required, thanks to the potential-energy bias introduced in the MetaD-WTE part; at the same time, the high-temperature replicas from the PT part may compensate for the limited number of CVs directly biased to explore a high-dimensional phase space. On the other hand, in the BEMD method, a larger set of structural CVs is separately biased, and the system is often simulated at one temperature. Overall, the efficiency of the CV-based metadynamics methods depends on a suitable set of CVs, which usually requires a priori knowledge of topological, chemical, and physical properties of the protein of interest. In this context, we have selected structural CVs commonly used for exploring folded and disordered proteins.^39,47,66^

It is well-known that the results of MD simulations can strongly depend on the accuracy of the applied protein–water force fields, which may lead to large discrepancies with various experimental measurements.^43,46,50,76−84^ Examples have been observed with state-of-the-art AMBER force fields a99SB*-ILDN^85,86^ with TIP3P,^87^ a99SB-ILDN with TIP4P-D,^82^ and the a03ws with empirically optimized solute–solvent dispersion interactions,^83^ CHARMM force fields CHARMM22*^88^ and CHARMM36m^89^ with CHARMM-modified TIP3P.^87,89,90^ For example, these force fields have been demonstrated to calculate very different helical propensities from the experimental estimates of NMR data for various proteins of interest.^76^ While the recently developed a99SB-disp is optimally parameterized for both folded and disordered proteins with substantially improved accuracy,^76^ the requirement of a four-point water model significantly increases computational costs; the force field is also reported to be too soluble for studying aggregations of some disordered proteins.^91,92^ Other important IDP-specific AMBER force fields include ff99IDPs,^93,94^ ff14IDPs,^94,95^ and ff14IDPSFF^94,96,97^ with the TIP3P water model,^87^ which were optimized by incorporating different backbone torsion parameters from different amino-acid groups including certain disorder-promoting amino acids. Here, we propose and demonstrate the use of two protein force fields compatible with three-point CHARMM-modified TIP3P water, namely, CHARMM36m and CHARMM22*, in combination with our chosen sampling methods. In particular, CHARMM36m showed improved accuracy in generating polypeptide backbone conformational ensembles for IDPs,^79,89^ despite the issue of over-compact structures and over-stabilized helices.^76,98^ The “helix-coil-balanced” CHARMM22*, instead, is not only able to reproduce experimental native-state structure and protein folding rate but also shows good agreement with experimental secondary structure propensities and NMR chemical shifts for many disordered proteins.^76,89,98^

In summary, we use BEMD and PTMetaD-WTE with CHARMM22* and CHARMM36m protein force fields to effectively sample IDP conformational landscapes within a microsecond time scale. We apply this approach to elucidate the structural and thermodynamic properties of an archetypal IDP sequence derived from the N-terminus of DEAD-box protein DHH1.^99,100^ DHH1N is a 46 amino-acid sequence that contains a low fraction of hydrophobic residues (Supplementary Note 1). The peptide is enriched in polar residues such as asparagine (Asn) and threonine (Thr), negatively charged aspartic acid (Asp), and positively charged large-sized arginine (Arg) and lysine (Lys), and with a few prolines (Pro) distributed along the C-terminal half of the peptide. Experimental observations indicate that in vitro DHH1N does not undergo LLPS on its own at physiological pH, although its composition is characteristic of LCDs involved in LLPS, and the fact that it participates in the formation of molecular adhesives to promote the LLPS of chimera proteins.^15^ In this work, we investigate a relatively short IDP that does not undergo phase separation, demonstrating how information from multiple sampling algorithms and force fields reveal a detailed and congruent multidimensional FES within microsecond time scales. This method represents a basis to next investigate the behavior of IDPs undergoing phase separation and connect structural properties with phase transition.

## Results and Discussion

### PTMetaD-WTE

**Figure 1:**
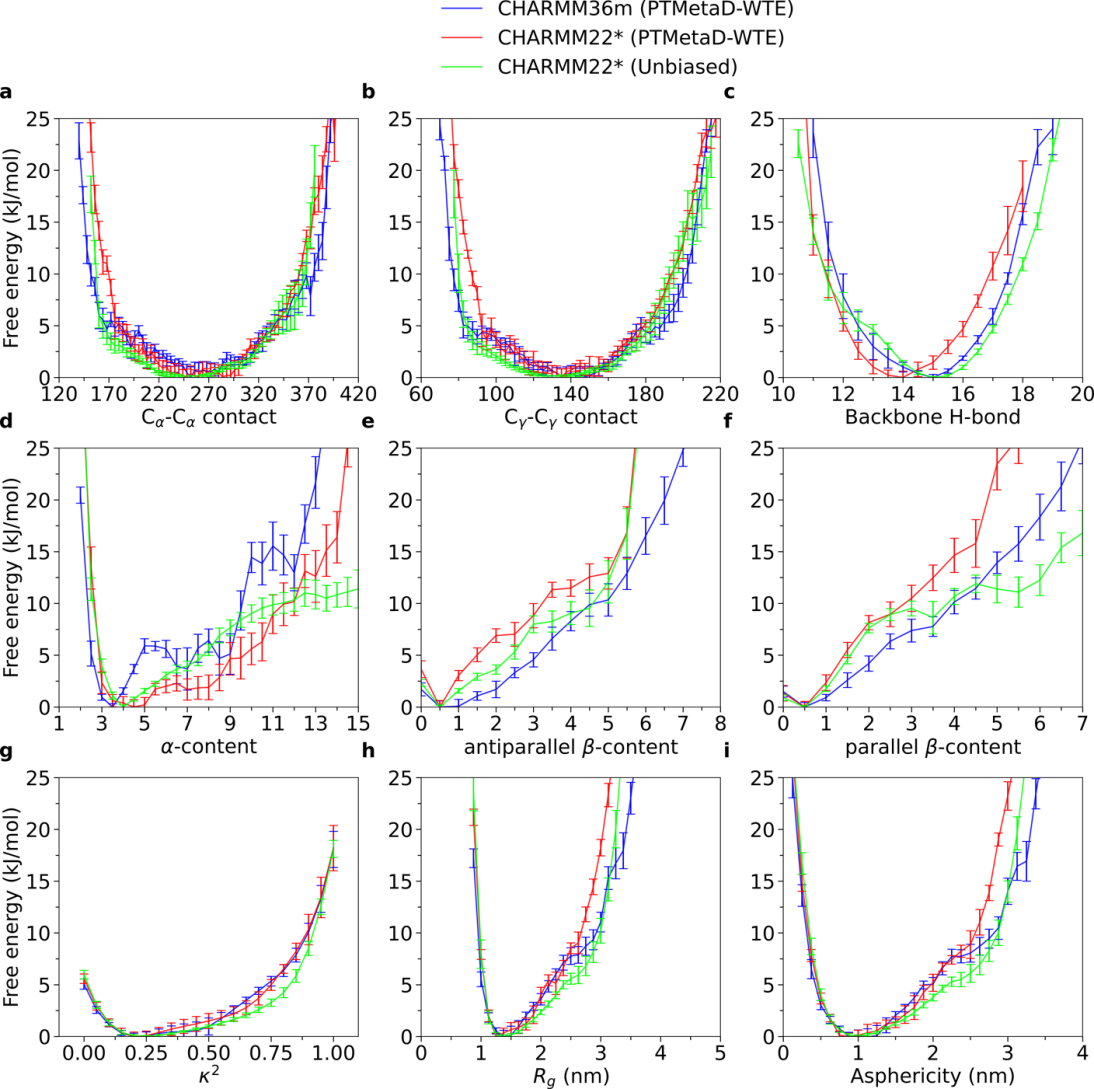
1D-FE profiles of DHH1N for CHARMM36m PTMetaD-WTE, CHARMM22* PTMetaD-WTE, and CHARMM22* unbiased simulations. Error bars are calculated from the Tiwary reweighting scheme^74,105^ and block averages.^103−105^

To obtain a general understanding of the configurational ensemble of DHH1N using PTMetaD-WTE, we first calculated one-dimensional FE (1D-FE) profiles as a function of individual CVs at 300 K for both CHARMM36m and CHARMM22* (Figure 1); in addition, results from the unbiased CHARMM22* simulations with nine different initial structures are also included for comparison. The selection of PLUMED-defined CVs includes the number of C_α_–C_α_ contacts and hydrophobic C_γ_*–*C_γ_ contacts, the number of backbone H-bonds, α-content, antiparallel-β-content and parallel-β-content, radius of gyration *R*_g_, asphericity *b*, and the relative-shape-anisotropy κ^2^, which measures the conformational deviation from a perfectly spherical structure (Supplementary Note 2).^101,102^ During PTMetaD-WTE, the number of C_α_–C_α_ contacts and hydrophobic C_γ_–C_γ_ contacts was explicitly biased. Generally, these CVs enable us to understand protein structures in terms of size, shape, compactness, and structural order.

**Figure 2:**
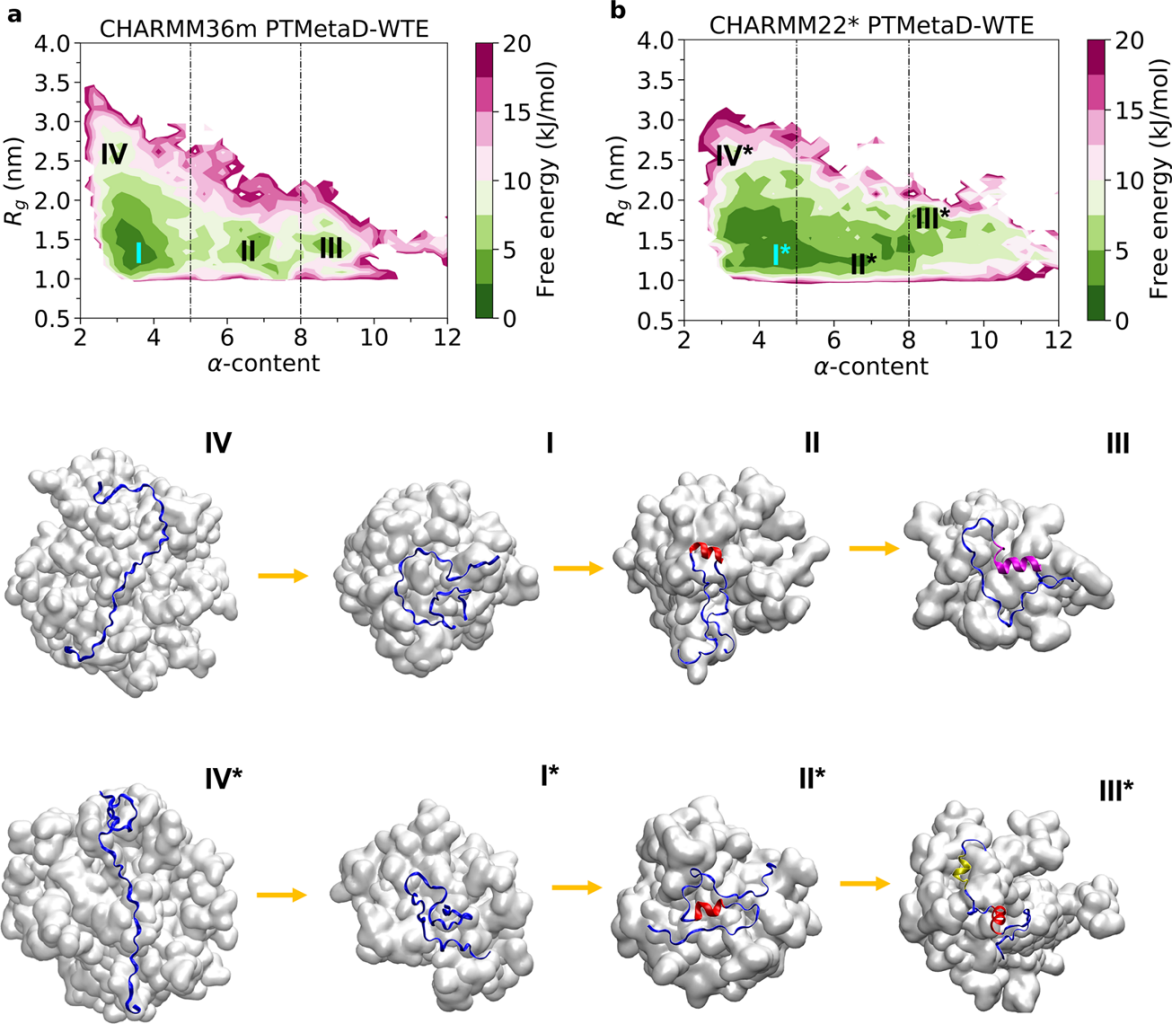
. 2D-FESs of DHH1N with PTMetaD-WTE on α-content and *R*_g_ for CHARMM36m (a) and CHARMM22* (b) with the black dashed lines indicating cluster 1 (α-content ≤ 5), cluster 2 (5 < α-content ≤ 8), and cluster 3 (α-content > 8). Conformations above 20 kJ/mol are not shown in the figure. Purple, red, and yellow parts of the protein highlight the dynamic helical regions of Asn5-Asp18, Asp18-Asp26, and Thr36-Thr42, respectively. The gray clouds represent the sampled conformational space of each annotated state.

Notably, the configurational ensemble projected in most CVs displays a single FE minimum upon convergence, corresponding to a monomodal probability density distribution (Supplementary Note 3 and Figures S1–S3). An exception is represented by the α*-*content distribution obtained with CHARMM36m, which shows a FE profile characterized by multiple local minima. A similar picture emerges, to a lesser extent, from the α-content probability density obtained from CHARMM22* PTMetaD-WTE, which also exhibits shallow local minima, despite a smoother FE profile compared to CHARMM36m. To investigate the possibilities of multiple, metastable conformational states emerging from PTMetaD-WTE simulations, we investigated conformational degeneracies associated with individual CVs by constructing two-dimensional FESs (2D-FESs) for pairs of CVs that are less correlated with each other (Supplementary Note 3 and Figure S4). Importantly, both force fields map rugged weakly funneled landscapes, with a single, large global basin at compact *R*_g_ and low α-content value (Figure 2). The local minima in the 1D-FE profile CHARMM36m (Figure 1d) are shown more clearly in the 2D-FES (Figure 2a), highlighting the existence of metastable basins associated with different conformational states separated by apparent FE barriers. Overall, CHARMM22* displays a conformational ensemble similar to that of CHARMM36m.

To probe DHH1N structures corresponding to different basins, we clustered the configurations sampled through PTMetaD-WTE into three groups. Cluster 1 contains all conformations with low α-content values (≤5), cluster 2 for all structures showing intermediate α-content values (>5 and ≤8), and eventually cluster 3 for all frames showing high α-content values (>8). Because the CV α-content correlates with the α-helix content by definition,^101^ conformations in cluster 3 may feature the highest amount of α-helices, while structures in cluster 1 are associated with the largest disorder. The global minima of CHARMM36m and CHARMM22* are both enclosed within cluster 1, which comprises approximately 70 and 50% of the equilibrium conformational population, respectively. Such evidence strongly indicates that DHH1N is intrinsically disordered regardless of the force-field choice, consistent with experimental observation.^15,99,100^ While the two force fields explore similar conformational phase space, no significant FE barriers are displayed between the clusters of CHARMM22*, suggesting that the force field may describe DHH1N with a higher conformational flexibility, and with lower FE costs for converting between compact and extended conformations. In contrast, the disordered global minimum of CHARMM36m is separated from the partially folded local minima by more apparent FE barriers, implying that the nucleation of α-helical domains is more likely to be an activated process for DHH1N simulated with CHARMM36m.

**Figure 3:**
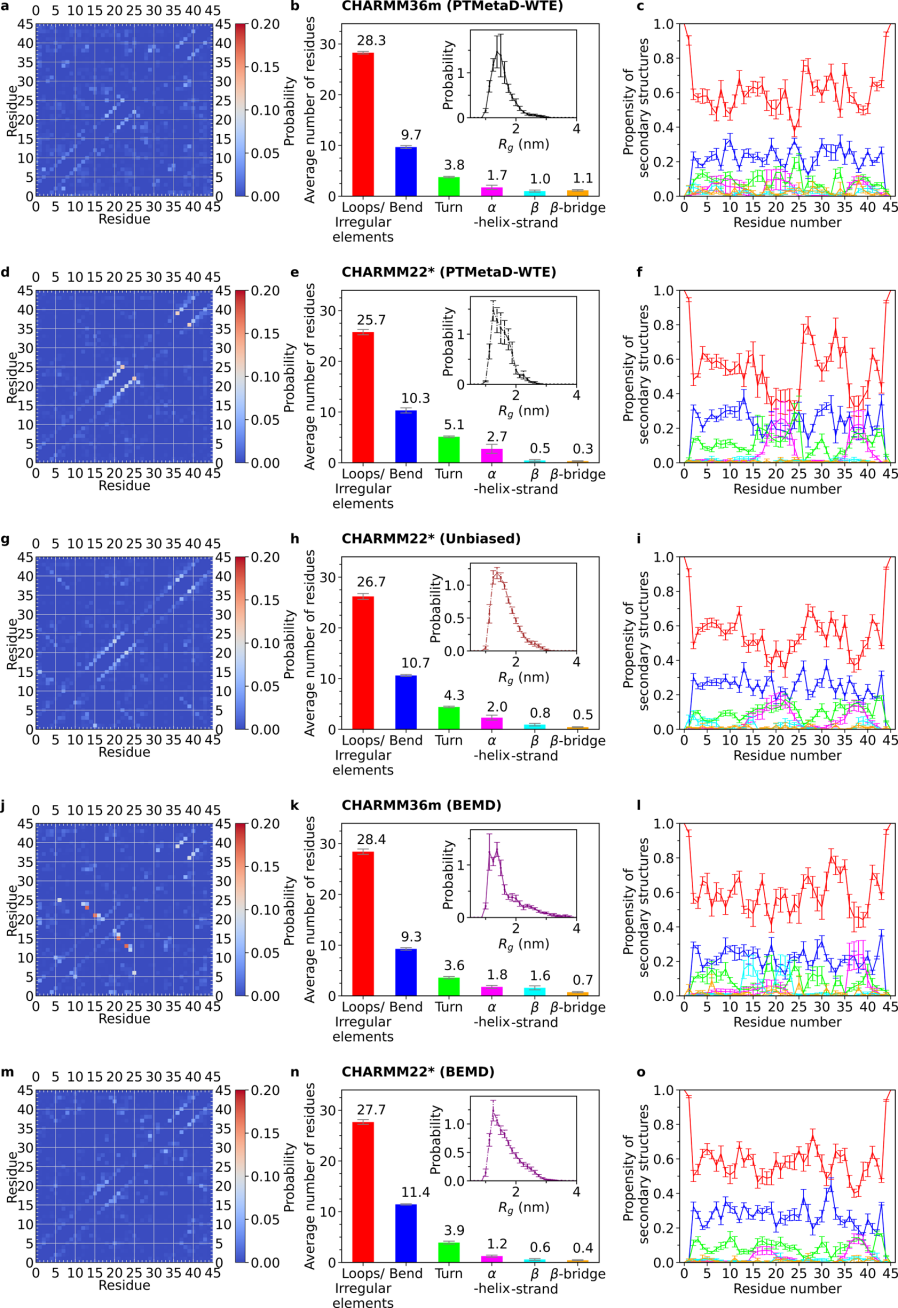
C_α_–C_α_ contact maps and secondary structure analysis of DHH1N for CHARMM36m PTMetaD-WTE (a–c), CHARMM22* PTMetaD-WTE (d–f), and CHARMM22* unbiased (g–i), CHARMM36m BEMD (i–j), and finally CHARMM22* BEMD (m–o). The insets of (b, e, h, k, and n) correspond to *R*_g_ distributions. The secondary structure assignment is based on the DSSP analysis codes.^106^ Error bars are calculated from the Tiwary reweighting scheme^74,105^ and block averages.^103−105^

To study more accurately the structural motifs emerging from the extensive sampling of the DHH1N conformational ensemble, we also evaluate the key secondary structure content for CHARMM36m and CHARMM22* PTMetaD-WTE by means of the DSSP algorithm.^106^ DHH1N is predominantly disordered, as demonstrated by the high number of loops/irregular elements, bends, and turns for both CHARMM36m and CHARMM22*. When modeled with CHARMM36m, DHH1N displays three domains with a relatively high propensity to nucleate α-helices, located approximately along the sequences of Asn6-to-Asp16, Asp18-to-Asn26, and Thr36-to-Thr42 (Figure 3c). Interestingly, the propensity of α-helices along the N-terminal Asn6-to-Asp16 is comparatively low compared with those of the other two regions, which becomes negligible in the case of CHARMM22* PTMetaD-WTE (Figure 3f) and unbiased simulation (Figure 3i), suggesting that DHH1N simulated under CHARMM22* demonstrates negligible probability to form α-helices in the Asn-rich N-terminal domain.

Moreover, the sequence between Pro28 and Pro34, which separates the α-helical domains of Asp18-to-Asn26 from Thr36-to-Thr42, shows no α-helix propensity for both CHARMM36m and CHARMM22* (Figure 3c,f,i). Such observation could be associated with the incompatibility of Pro with α-helix formation due to its rigid-ring structure and absence of a H atom on the peptide bond N necessary for further H-bonding.^107,108^ This feature is well captured by both force fields. To study individual residue–residue interactions in detail, we constructed C_α_–C_α_ contact maps for residue pairs beyond the next nearest neighbors using a threshold distance of 0.5 nm (Figure 3a,d,g). By focusing on residue–residue contacts with ∼5% frequencies, our analysis reveals a sparse contact map with only a few key contacts along the map diagonal for all of CHARMM36m/CHARMM22* PTMetaD-WTE/unbiased simulations. The adjacent contacts along the diagonal in the domains of Asp18-to-Asn26 and Thr36-to-Thr42 coincide approximately with the two helical regions for both CHARMM36m and CHARMM22* from DSSP analysis (Figure 3c,f,i).

### Bias-Exchange Metadynamics

**Figure 4:**
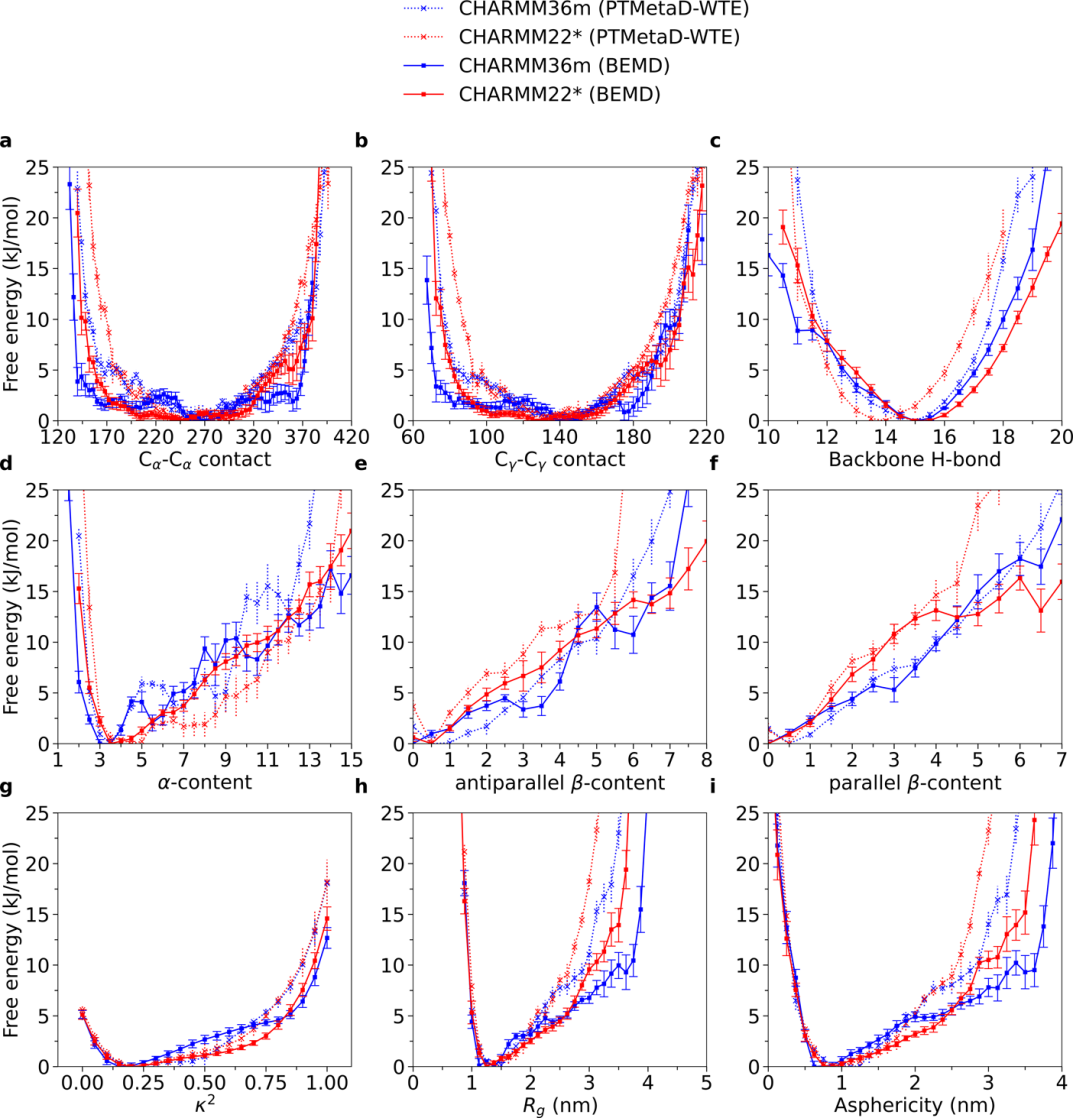
Comparison between the 1D-FE profiles of DHH1N from PTMetaD-WTE and BEMD simulations. Error bars are calculated from the Tiwary reweighting scheme^74,105^ and block averages.^103−105^

To complement the insights obtained with PTMetaD-WTE, we adopted the BEMD method to further explore the conformational ensemble of DHH1N. We constructed 1D-FE profiles for CHARMM36m and CHARMM22* at 300 K and compare them with the results from PTMetaD-WTE. Similarly, the configurational ensemble projected in most CVs also displays a single FE minimum (Figure 4), corresponding to a monomodal probability density distribution upon convergence (Supplementary Note 4 and Figures S5, S6). All our simulations show that DHH1N contains a very low amount of β-sheet (parallel/antiparallel β-content) and α-helical (α-content) motifs, featuring the intrinsically disordered nature of the protein (Figure 4d–f). However, BEMD tends to explore wider ranges of C_α_–C_α_ and C_γ_*–*C_γ_ contacts than PTMetaD-WTE (Figure 4a,b) (Supplementary Note 5 and Figure S7a,b), with the effect being more prominent for CHARMM36m. For CHARMM36m, BEMD agrees well with PTMetaD-WTE on producing α-content FE profiles characterized by multiple local minima (Figure 4d) (Supplementary Note 5 and Figure S7d), despite the fact that some of the local minima are located at different α-content values. In addition, the incapability of DHH1N to undergo LLPS may also be reflected in its relative-shape-anisotropy κ^2^. The broad distribution of κ^2^ in all four simulations (Supplementary Note 5 and Figure S7g) suggests that multiple DHH1N molecules may dynamically adopt different molecular shapes because their conformations are not separated by large FE barriers.

**Figure 5:**
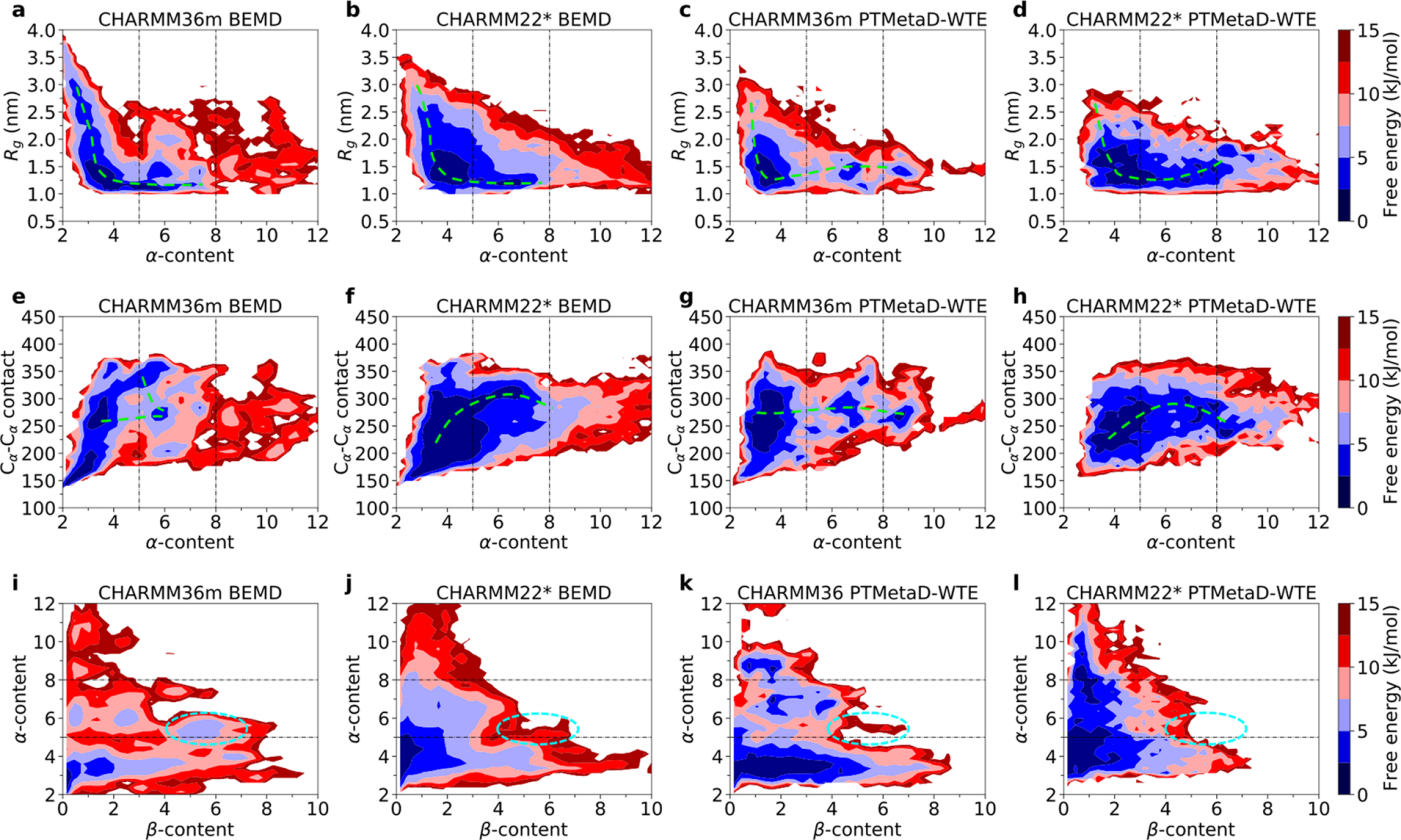
2D-FESs on α-content and *R*_g_ for CHARMM36m BEMD (a), CHARMM22* BEMD (b), CHARMM36m PTMetaD-WTE (c), and CHARMM22* PTMetaD-WTE (d). 2D-FESs on α-content and the number of C_α_–C_α_ contact for CHARMM36m BEMD (e), CHARMM22* BEMD (f), CHARMM36m PTMetaD-WTE (g), and CHARMM22* PTMetaD-WTE (h). 2D-FESs on β-content and α-content for CHARMM36m BEMD (i), CHARMM22* BEMD (j), CHARMM36m PTMetaD-WTE (k), and CHARMM22* PTMetaD-WTE (l). The black dashed lines indicate clusters 1 (α-content ≤ 5), 2 (5 < α-content ≤ 5), and 3 (α-content > 8) as defined in Figure 2. The green dashed lines are schematic representation of possible pathways between the α-content minima. The cyan dashed lines indicate the regions primarily occupied by the subgroup of CHARMM36m BEMD with concurring Ala24 and Ser16-Lys22 contacts, which is less explored in all the other simulations.

To further examine the conformational states of DHH1N, we construct a 2D-FES for CHARMM36m and CHARMM22* BEMD and compare them with the 2D-FES from PTMetaD-WTE. Similarly, every simulation reveals a global minimum at low α-content in the disordered cluster 1, accompanied by multiple shallow local minima, indicating a large number of substates as a result of conformational heterogeneity (Figure 5). Cluster 1 of CHARMM36m and CHARMM22* BEMD comprises approximately 70% of the equilibrium conformational population, similar to the result of CHARMM36m PTMetaD-WTE, while cluster 1 population of CHARMM22* PTMetaD-WTE is much lower due to the relatively shorter simulation time (Supplementary Note 5 and Table S3). The largest disparities between the 2D-FES are primarily due to different choices of force fields. In detail, the CHARMM36m results show that the nucleation of local α-helical domains is an activated process for both PTMetaD-WTE and BEMD, as suggested by the presence of relatively large FE barriers (Figure 5a,c,e,g,i,k), despite the fact that the distribution of local minima differs slightly between the two sampling methods. Conversely, the assembly/disassembly of the partially ordered domains in CHARMM22* is associated with relatively small FE costs, as indicated by a reduced number of scattered local minima and FE barriers for both sampling methods (Figure 5b,d,f,h,j,l). It is challenging to further compare the details of the scattered local minima because the weakly funneled and rugged nature of the FE landscape means that statistical errors could be of similar magnitude.

Based upon the 2D-FE analysis, we proceeded contact map and secondary structure analysis for CHARMM36m and CHARMM22* of BEMD, in comparison with the results from CHARMM36m and CHARMM22* of PTMetaD-WTE (Figure 3). By focusing on residue–residue contacts with ∼5% frequencies, our analysis again reveals a sparse contact map in which key contacts are mostly along the diagonal regions for all simulations. In respect of the off-diagonal regions, the four simulations show a lack of common residue contacts. One exception is the Asn14-Ala24/Ser16-Lys22 domain of CHARMM36m BEMD. The contact map of CHARMM36m BEMD displays prominent Asn14-Ala24 and Ser16-Lys22 contacts in this region, which is noticeably different from the other simulations within the microsecond simulation length (Figure 3i). At the same time, CHARMM36m BEMD explores a relatively lower number of α-helical structures and a comparatively higher amount of β-strands in the domain of Asp18-to-Asn26, compared to the other method/force-field combinations (Figure 3l). To investigate possible relationship between the two features, we extracted structures with concurring Asn14-Ala24 and Ser16–Lys22 contacts from the CHARMM36m BEMD configurational ensemble and found that these residue contacts are correlated with the presence of β-strands within Asn14-to-Asn26 (Supplementary Note 6 and Figures S8, S9). The subgroup occupies ∼18% of the CHARMM36m BEMD population and spans both clusters 1 and 2 (Supplementary Note 6 and Figure S9b) (Figure 5). The relevant conformations, highlighted by the cyan circles in Figure 5i, are less explored in the other force-field/method combinations (Figure 5j–l).

**Figure 6:**
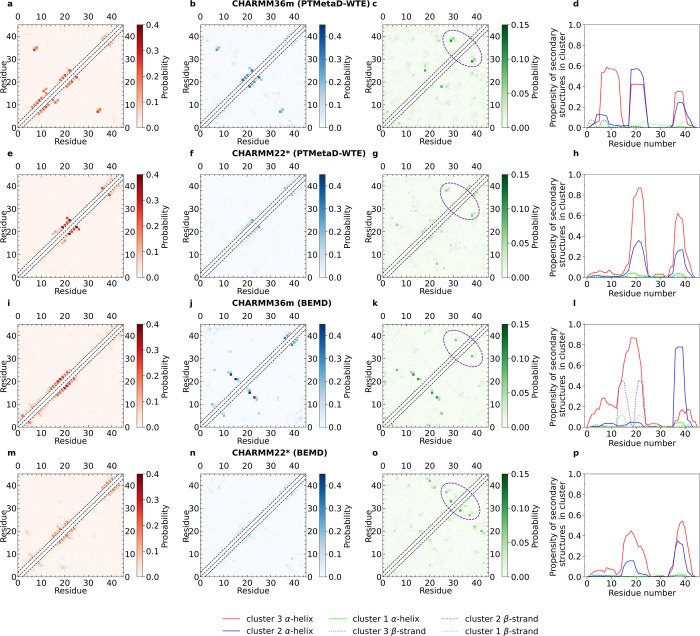
C_α_–C_α_ contact map and α-helical and β-strand analysis of DHH1N of clusters 1, 2, and 3 for CHARMM36m PTMetaD-WTE (a–d), CHARMM22* PTMetaD-WTE (e–h), CHARMM36m BEMD (i −l), and CHARMM22* BEMD (m–p). The probabilities and propensities are computed relative to the overall population of every cluster. The purple dashed lines highlight possible regions of residues contacts (approximately between Pro28-Thr32 and Pro34-Leu40) that are shared by all simulations.

We also constructed C_α_–C_α_ contact maps and secondary structure analysis for clusters 1, 2, and 3 of all simulations (Figure 6). For CHARMM36m BEMD, the concurring Asn14-Ala24 and Ser16-Lys22 contacts, which have been proven to be highly associated with the β-strands observed along Asn14-to-Asn26, emerge primarily in the less-ordered clusters 1 and 2 (Figure 6j,k). In all cases, adjacent diagonal residue contacts are focused within the two highest-order clusters (2 and 3), reflecting the presence of locally ordered domains for the generally disordered DHH1N. In contrast, the four force-field/method combinations share very few off-diagonal contacts, especially in the largest and most-disordered cluster 1, which contains about 70% of overall population in most cases. A potential region of common contacts can be approximately between Pro28-Thr32 and Pro34-Leu40 (Figure 6). The first sequence is capped by Pro28 and Pro34 and contains two adjacent units of positively charged and bulky Lys29-Lys30), while the second sequence contains two adjacent units of negatively charged Asp37-Asp38. Thus, electrostatic interactions may contribute to forming these residue contacts, and it is also possible that participating in α-helical motifs reduces the likelihood of Pro34-Leu40 to interact with Pro28-Thr32 in clusters 2 and 3.

Overall, CHARMM36m BEMD shows a tendency to sample more β-sheet-displaying conformations than the other force-field/method combinations. As shown earlier, a large fraction of the relevant structures corresponds to forming antiparallel β-sheets along Asp18-to-Asn26 and α-helices along Thr36-to-Thr42, with concurring Asn14-Ala24 and Ser16-Lys22 residue contacts (Supplementary Note 5, Figure S7 and Supplementary Note 6, Figures S8, S9). Such trend is not observed for CHARMM22* simulated under the BEMD method and CHARMM36m simulated under PTMetaD-WTE. One hypothesis could be that the feature could be specifically related with using the backbone-optimized force field in combination with the sampling method. In the literature, it is also reported that BEMD explores a larger portions of phase space than simple temperature PT simulations.^43^ Our evidence also shows that BEMD is able to sample a slightly larger conformational ensemble than PTMetaD-WTE (Figure S7a,b). Thus, it is possible that the observed discrepancy can be due to the fact that both α-content and β-content were used as CVs in BEMD, while the bias potential of PTMetaD-WTE (a function of C_α_–C_α_ and C_γ_–C_γ_ contacts) can only implicitly explore along the two secondary structure reaction coordinates. Nevertheless, the difference may be relatively trivial in the presence of the dominating disordered nature of DHH1N—over 70% of the total population is contained in the low β-content (β-content ≤ 4) region for both CHARMM36m BEMD and PTWTE. Furthermore, all simulations provide a similar insight into a possible nucleation pathway for the partially disordered regions of DHH1N (schematically represented by the green dashed lines in Figure 5). While the 2D-FES cannot reveal an accurate multidimensional pathway for the full conformational transition kinetics,^109^ which could be a common challenge for representing protein FEs in the low-dimensional space of popular CVs with physical meanings, our evidence indicates that the dynamic disorder → α transition in the local domains of Asp18-to-Asn26 and Thr36-to-Thr42 is likely to occur within a relatively compact DHH1N (*R*_g_ ∼ 1.5 nm) with moderate sphericity (Figure 5 and Supplementary Note 5, Figure S7g–i). Nevertheless, the high conformational heterogeneity of IDPs means that the exact locations and energies of the metastable states and barriers would require a more accurate FE representation of reduced dimensionality.

## Conclusions

In this work, we have examined the use of different protein force-field/sampling method combinations to obtain a consensus picture for the structural and thermodynamic features of the disordered sequence DHH1N at ambient temperature and physiological pH. Despite different fine details, we show that the conformational landscapes emerging from different force-field/sampling method combinations are largely congruent. While we do observe discrepancies in some properties from separate simulations, all results show good agreement in multidimensional FESs, general features of residue–residue contact map and secondary structure analysis, which are consistent with the disordered nature of the protein. The power of modern computers has increased dramatically, but many explicit-solvent protein simulations are still limited to the microsecond time scale, which adds to the need for better sampling schemes beyond purely unbiased simulations. In this context, DHH1N is a relatively short 46-amino-acid IDP and a comparison between biased and unbiased simulations is conducted for the more flexible CHARMM22* force field, so the time scale of unbiased conformational transitions (starting from a few initial configurations) becomes comparable to those of the biased methods (Supplementary Note 10 and Figures S13, S14); nevertheless, the fact that the averages of the combined unbiased data agree with the biased results is probably associated with a lucky choice of initial structures. Some unbiased simulations can show large fluctuation/relaxation time, reduced reversibility, or conformational ensembles that are only converged locally (Supplementary Note 10 and Figures S13, S14).

As a result of the specific features of various protein force fields and sampling methods, different portions of phase space may be preferably explored within finite simulation time, despite the fact that all methods should converge in the long-time limit. Hence, relying on the results from a single force-field/sampling method may restrain our understanding of the protein conformational landscape. For example, the temperature dependence of certain protein properties can be directly recovered by analyzing the sampling obtained from PTMetaD-WTE, which can provide key structure–property information to help understand protein phase behavior at different temperatures.^110^ On the other hand, BEMD simulations directly explore more CVs and possibly larger portions of phase space within a finite simulation time, which can be preferred if certain metastable states can be efficiently explored by enhancing the sampling along specific CVs. Hence, in order to develop a comprehensive picture, it may be beneficial to include multiple FF/sampling method combinations in the study of IDPs. For example, PTMetaD-WTE with multiple CV combinations could also be conducted to compare the conformational space sampled within the same computational timeframe.

The evidence gathered from our study shows that DHH1N has an average *R*_g_ between 1.54 and 1.64 nm (Table 1), which is comparable with that of the other phase-separating IDPs with a significantly longer sequence.^23^ Such feature indicates a relatively low level of compactness. In the literature, it is reported that the phase-separating behavior of disordered proteins can be generally associated with their single-chain compactness, and the sequence determinant of the compaction of disordered proteins is not only related to the overall protein charge but also affected by the organization of charged and aromatic residues along the peptide sequence.^24,29,32,111−114^ At physiological pH, DHH1N is electrostatically neutral, but its charged residues, that is, negatively charged Asp and positively charged Lys and Arg, are mostly focused within the C-terminal portion of the sequence from Asp16 onward. However, DHH1N does not contain high-probability off-diagonal residue contacts that are shared by all force-field/method combinations, implying an absence of salt bridges or favorable electrostatic attractions between oppositely charged patches, which could be key factors in inducing complex coacervation of many phase-separating IDPs.^21,23^ Scarce presence of aromatic amino acids in DHH1N also means that the protein is not likely to form condensates stabilized by cation−π and π–π interactions.

**Table 1:** Average Radius of Gyration for DHH1N; Errors Are Calculated from Block Analysis

	CHARMM36m PTMetaD-WTE	CHARMM22* PTMetaD-WTE	CHARMM22* BEMD	CHARMM36m BEMD
⟨*R*_g_⟩ (nm)	1.55(0.03)	1.54(0.02)	1.66(0.04)	1.65(0.04)

The comprehensive approach implemented in this work, which we have initially applied to analyze a relatively short IDP that does not undergo phase separation in vitro, represents a prospecting platform to investigate the conformational ensemble and thermodynamic driving force of other IDPs that exhibit liquid–liquid phase separation under a broad range of solution conditions. Such analysis will provide crucial information at the atomistic level which will be key to unravel biological phase separation in general and assist the design of new building blocks for advanced protein-based materials and microreactors.

## Methods

### System Preparation and Equilibration

Nine initial guess configurations for the folded protein structure were obtained from i-TASSER^115^ and Robetta webservers^116^ (Supplementary Note 7). Force-field parameters were assigned by means of the *pdb2gmx* command implemented in GROMACS 2019.3 software;^117^ protonation states were assigned assuming physiological pH 7. The overall charge of the protein was equal to 0 and no counterions or salts were added to assure electroneutrality. CHARMM22*^88^ and CHARMM36m^89^ force fields were chosen for the protein, while the CHARMM-modified TIP3P model^87,89,90^ was used for water. The initial protein structure was placed in the center of the simulation box and subsequently solvated through *editconf* and *solvate* commands implemented in GROMACS 2019.3 software. All simulations were conducted using GROMACS 2019.3 patched with PLUMED 2.5.2.^118^ Broadly, system equilibration was carried out according to the following protocol. First, system minimization was performed using the steepest descent algorithm, using a tolerance value of 1000 kJ mol^–1^ nm^–1^ for the force. Temperature was raised to and kept at the target value for 50 ps in the *NVT* ensemble (*N*: number of particles; *V*: volume; *T*: temperature) by means of the V-rescale algorithm.^119^ Solvent density was subsequently equilibrated in the *NpT* ensemble (*N*: number of particles; *p*: pressure; *T*: temperature), adopting the Parrinello–Rahman barostat^120^ to keep the pressure to the target value of 1 atm. Neighbor list was updated every 10 simulation steps using a Verlet cutoff scheme.^121^ Electrostatic long-range interactions were computed by particle mesh Ewald^122^ using a cutoff value equal to 1.0 and 1.2 nm for CHARMM22* and CHARMM36m force fields, respectively; the same cutoff values were employed for van der Waals (vdW) interactions. In more detail, for CHARMM36m, we adopted a force-switch scheme for vdW interactions as suggested by GROMACS, setting *rvdw-switch* equal to 1.0 nm.^123^ The LINCS algorithm^124^ was employed to constrain all covalent bonds involving hydrogen atoms, which allowed a time step of 2 fs to propagate system dynamics via the Leap-Frog algorithm. All simulations were performed adopting periodic boundary conditions (PBCs). Further details of simulations are included in Supplementary Notes 8 and 9.

### Parallel-Tempering Well-Tempered Metadynamics

Structure 1 of the nine initial guess configurations was first equilibrated in the *NpT* ensemble for approximately 5 ns using the V-rescale algorithm^119^ and the Parrinello–Rahman barostat^120^ to keep the temperature and pressure at ambient conditions. After this, the equilibrated structure was used as the starting configuration for PTMetaD-WTE^39,47,68−70^ simulations for both CHARMM36m^89^ and CHARMM22*.^88^ In the first stage, PTMetaD-WTE was implemented biasing only the potential-energy (PE) CV of each temperature replica, simulating at *T* = 300, 308, 317, 326, 335, 345, 354, 364, 374, 385, 396, 407, 418, 430, 442, 455, 467, 481, 494, 508, 522, 537, 552, and 568 K for 20 ns per replica. The accumulated bias potential was subsequently used as a static PE bias potential in the second stage, where alpha carbon C_α_**–**C_α_ and gamma carbon hydrophobic C_γ_**–**C_γ_ contacts were both biased according to the well-tempered metadynamics algorithm. The definition and input parameters of the CVs are included in Supplementary Note 2. The bias-factor for PE CV and the two conformational CVs were both 12, with initial Gaussian widths of 2000 kJ mol^–1^ and 1.0, respectively, at a Gaussian height of 1.2 kJ mol^–1^. A total of 1000 ns of data per temperature replica were used to reconstruct the FES of DHH1N at *T* = 300 K. A similar protocol was applied to CHARMM22* PTMetaD-WTE to obtain approximately 600 ns of data. We used an exchange frequency of every 500 steps with acceptance probabilities between 8 and 20%. The PLUMED input files required to reproduce the PTMetaD-WTE simulations are available on PLUMED-NEST (www.plumed-nest.org), the public repository of the PLUMED consortium,^125^ under the project ID plumID:21.036.

### Bias-Exchange Metadynamics

BEMD^66,67^ simulations were carried out adopting seven CVs (the number of C_α_–C_α_ contacts, C_γ_–C_γ_ hydrophobic contacts, and backbone H-bonds, dihedral correlation, α-content, antiparallel β-content, and parallel β-content, as defined in Supplementary Note 2) and eight replicas, with one CV per replica plus the unbiased replica. One of the initial guess configurations was first equilibrated in the *NpT* ensemble at 1 atm and 300 K for about 5 ns per replica in the *NpT* ensemble according to the discussed protocol (vide supra); BEMD simulations were performed in the *NVT* ensemble at 300 K, collecting 1000 ns of data for each replica; exchange of conformations between two randomly selected replicas was periodically attempted every 10,000 simulation steps. CV was biased according to the ordinary metadynamics scheme, adding the bias potential every 2500 simulation steps using a height value equal to 0.3 kJ mol^–1^. After 160 ns, the system explored a wide region for each CV and we introduced loose lower and upper boundaries to improve convergence (Supplementary Note 8). The PLUMED input files required to reproduce the BEMD simulations are available on PLUMED-NEST (www.plumed-nest.org), the public repository of the PLUMED consortium,^125^ under the project ID plumID:21.036.

### Unbiased Simulations

Unbiased simulations were performed adopting only the CHARMM22*^88^ force field. Nine of the input guess structures were solvated and equilibrated at 1 atm and 300 K according to the discussed protocol (vide supra); 1000 ns MD simulations were subsequently performed in the *NVT* ensemble at 300 K for each system. The nine sets of data were concatenated for data analysis.

### Analysis of Simulation Data

All data analysis and error estimation are included in Supplementary Note 10.

## Supporting Information Available

Amino acid sequence for DHH1N; Collective variables definition and input parameters; Figure S1: One-dimensional probability densities of DHH1N as a function of single collective variables at 300 K with CHARMM36m PTMetaD-WTE, CHARMM22* PTMetaD-WTE and CHARMM22* unbiased simulations; Figure S2: Time evolution of the one-dimensional probability densities of DHH1N as a function of single collective variables at 300 K for CHARMM36m PTMetaD-WTE; Figure S3: Time evolution of the one-dimensional probability densities of DHH1N as a function of single collective variables at 300 K for CHARMM22* PTMetaD-WTE; Figure S4: Correlation of the collective variables for DHH1N; Figure S5: Time evolution of the one-dimensional probability densities of DHH1N as a function of single collective variables at 300 K for CHARMM36m BEMD; Figure S6: Time evolution of the one-dimensional probability densities of DHH1N as a function of single collective variables at 300 K for CHARMM22* BEMD; Figure S7: One-dimensional probability densities of DHH1N as a function of single collective variables at 300 K for CHARMM36m PTMetaD-WTE, CHARMM22* PTMetaD-WTE, CHARMM22* unbiased, CHARMM36m BEMD, and CHARMM36m BEMD simulations; Table S3: Fraction of total equilibrium population (%) estimated from the histogram probability densities in Figure S7d; Figure S8: Contact-map and secondary-structure analysis for all conformations with concurring Asn14-Ala24 and Ser16-Lys22 contacts for CHARMM36m BEMD; Figure S8: Contact-map and secondary-structure analysis for all conformations with concurring Asn14-Ala24 and Ser16-Lys22 contacts for CHARMM36m BEMD; Figure S9: Representative structure for the subgroup with concurring Asn14-Ala24 and Ser16-Lys22 contacts from CHARMM36m BEMD; Figure S10: Nine initial structures for CHARMM22* unbiased simulations, built from i-Tasser and Robetta; Input parameters for bias-exchange metadynamics simulations; Table S4: Input parameters of BEMD and CHARMM22* force field; Table S5: Input parameters of BEMD and CHARMM36m force field; Summary of performed simulations; Data analysis and error estimate (PDF)
