# Supplementary material for: Modeling the Structure and Interactions of Intrinsically Disordered Peptides with Multiple Replica, Metadynamics-Based Sampling Methods and Force-Field Combinations
Source: J Chem Theory Comput. 2022 Feb 17;18(3):1915–28. doi: 10.1021/acs.jctc.1c00889 (PMC9097291; doi:10.1021/acs.jctc.1c00889)
Supplement: Supplementary file 1 — ct1c00889_si_001.pdf [file ct1c00889_si_001.pdf]

Supporting Information for:

"Modelling the structure and interactions of intrinsically disordered peptides with multiple-replica, metadynamics-based sampling methods and force-field combinations"

Lunna Li[1,‡], Tommaso Casalini[2,‡], Paolo Arosio[2] and Matteo Salvalaglio[1*]

[1]*Thomas Young Centre and Department of Chemical Engineering, University College London, London WC1E 7JE, United Kingdom*
[2]*Department of Chemistry and Applied Biosciences, ETH Zurich, 8093 Zurich, Switzerland*

Supplementary Note 1:

Amino acid sequence for DHH1N[1-2]

GLY1– SER2 – ILE3 – ASN4 – ASN5 – ASN6 – PHE7 – ASN8 – THR9 – ASN10 – ASN11 – ASN12 – SER13 – ASN14 – THR15 – ASP16 – LEU17 – ASP18 – ARG19 – ASP20 – TRP21 – LYS22 – THR23 – ALA24 – LEU25 – ASN26 – ILE27 – PRO28 – LYS29 – LYS30 – ASP31 – THR32 – ARG33 – PRO34 – GLN35 – THR36 – ASP37 – ASP38 – VAL39 – LEU40 – ASN41 – THR42 – LYS43 – GLY44 – ASN45 – THR46

Supplementary Note 2

Collective variables definition and input parameters

$C_\alpha - C_\alpha$ contacts, $C_\gamma - C_\gamma$ contacts and the number of backbone H-bonds were computed by means of COORDINATION collective variable implemented in PLUMED[3]. The number of contacts N_c between two groups of atoms (A and B) is defined as follows:

$$N_c = \sum_{i \in A} \sum_{j \epsilon B} s_{ij} \tag{S1}$$

where the index i spans the atoms in the group A and the index j spans the atoms in the group B; s_{ij} is a switching function (which varies between 0 and 1) defined as follows:

$$s_{ij} = \frac{1-\left(\frac{r_{ij}-d_0}{r_0}\right)^n}{1-\left(\frac{r_{ij}-d_0}{r_0}\right)^m} \tag{S2}$$

where r_{ij} is the distance between the i atom of group A and the j atom of group B and d_0, r_0, n and m are parameters of the switching function. Group A and group B are both constituted by C_α and C_γ atoms for $C_\alpha - C_\alpha$ contacts and $C_\gamma - C_\gamma$ contacts, respectively; group A accounts for backbone hydrogen atoms and group B includes backbone oxygen atoms for the calculation of backbone H-bonds. Input parameters are summarized in Table S.1.

Table S.1. Input parameters of switching function for $C_\alpha - C_\alpha$ contacts, $C_\gamma - C_\gamma$ contacts and the number of backbone H-bonds.

**Collective variable**	**r_0 [nm]**	**d_0**	**n**	**m**
$C_\alpha - C_\alpha$ contacts	0.65	0	8	10
$C_\gamma - C_\gamma$ contacts	0.5	0	8	10
Backbone H-bonds	0.2	0	8	10

To improve the computational efficiency, we employed a neighbour list (NLIST command in PLUMED) which was updated every 5 simulation steps (NL_STRIDE command) and adopting a cutoff value equal to 5, 4 and 3 nm for $C_\alpha - C_\alpha$ contacts, $C_\gamma - C_\gamma$ contacts and the number of

backbone H-bonds, respectively (NL_CUTOFF command). Such values were chosen so that for r_{ij} values equal or higher than the cutoff, s_{ij} was constant and equal to 0.

Dihedral correlation d_c was computed with respect to ψ backbone dihedral angles by means of DIHCOR collective variable implemented in PLUMED:

$$d_c = \frac{1}{2}\sum_{i=2}^{N_d}[1 + \cos(\psi_i - \psi_{i-1})] \tag{S3}$$

where N_d is the number of considered dihedral angles.

The alpha helical, parallel, and antiparallel beta sheet content in the protein were obtained by means of ALPHARMSD, PARABETARMSD and ANTIBETARMSD collective variables implemented in PLUMED and proposed by Pietrucci and Laio[4]. The collective variable has the following form:

$$s = \sum_i \frac{1-\left(\frac{r_i-d_0}{r_0}\right)^n}{1-\left(\frac{r_i-d_0}{r_0}\right)^m} \tag{S4}$$

where the index i spans all possible groups of six residues that can form an alpha helix, a parallel beta sheet or an antiparallel beta sheet and r_i is the root mean square displacement (RMSD) between the actual structure of the group and an ideal alpha helix, parallel beta sheet or antiparallel beta sheet; d_0, r_0, n and m are parameters of the switching function. Input parameters are summarized in Table S.2.

Table S.2. Input parameters of switching function for alpha helical content, antiparallel beta sheet content and parallel beta sheet content.

Collective variable	r_0 [nm]	d_0	n	m
α-RMSD	0.08	0	2	4
Antiparallel β-RMSD	0.08	0	8	12
Parallel β-RMSD	0.08	0	8	12

Mass-weighted radius of gyration R_g, asphericity b' and the relative shape anisotropy κ^2 were calculated using the GYRATION collective variable implemented in PLUMED[1], whose definitions were originally proposed by Vymětal and Vondrášek[3].

The radius of gyration was calculated using:

$$s_{Gyr} = \left(\frac{\sum_i^n m_i |r_i - r_{\mathrm{COM}}|}{\sum_i^n m_i}\right)^{1/2} \quad \text{(S5)}$$

where n is the number of atoms in the protein, with the position of the centre of mass r_{COM} given as:

$$r_{\mathrm{COM}} = \frac{\sum_i^n r_i m_i}{\sum_i^n m_i} \quad \text{(S6)}$$

The asphericity was calculated using:

$$b' = \left[S_1 - \frac{1}{2}(S_2 + S_3)\right]^{1/2} \quad \text{(S7)}$$

The relative shape anisotropy was calculated using:

$$\kappa^2 = 1 - 3\frac{S_2 + S_2 S_3 + S_1 S_3}{(S_1 + S_2 + S_3)^2} \quad \text{(S8)}$$

where I_1, I_2 and I_3 are the three eigenvalues of the gyration tensor:

Supplementary Note 3

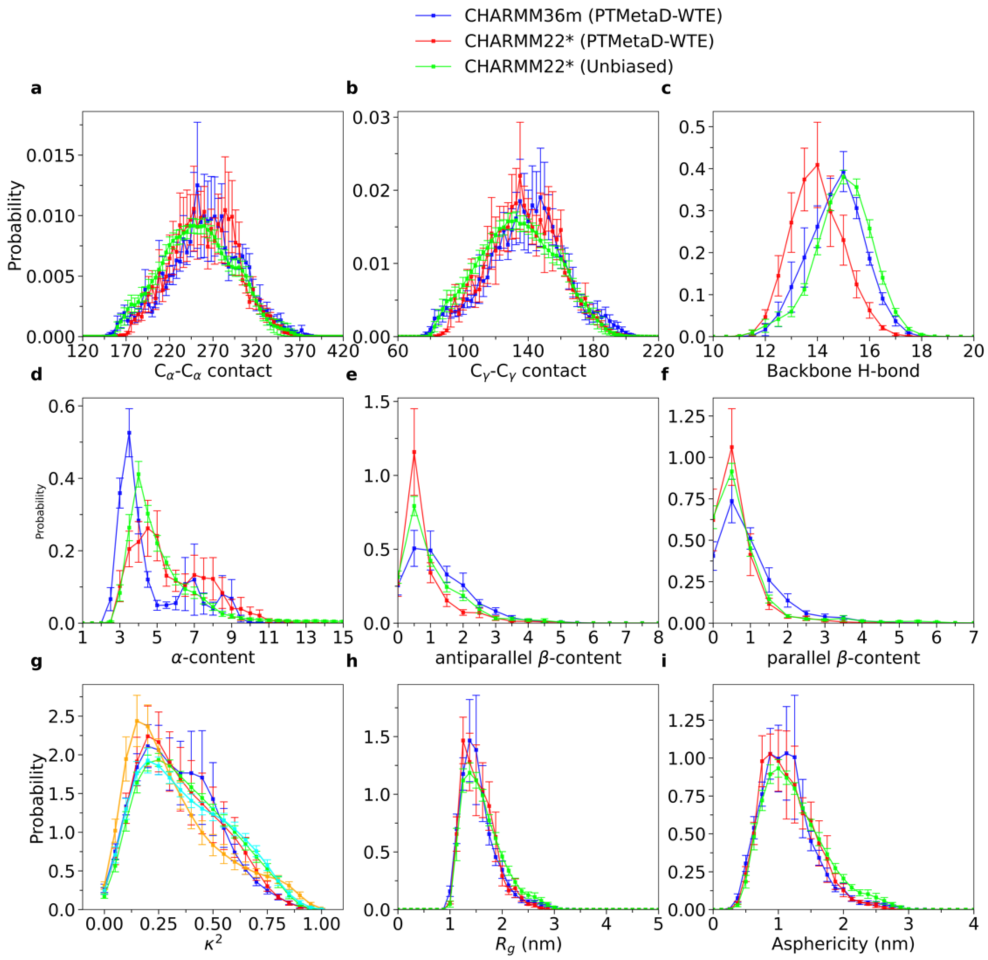

Figure S1 One-dimensional probability densities of DHH1N as a function of single collective variables at 300 K with CHARMM36m PTMetaD-WTE, CHARMM22* PTMetaD-WTE and CHARMM22* unbiased simulations.

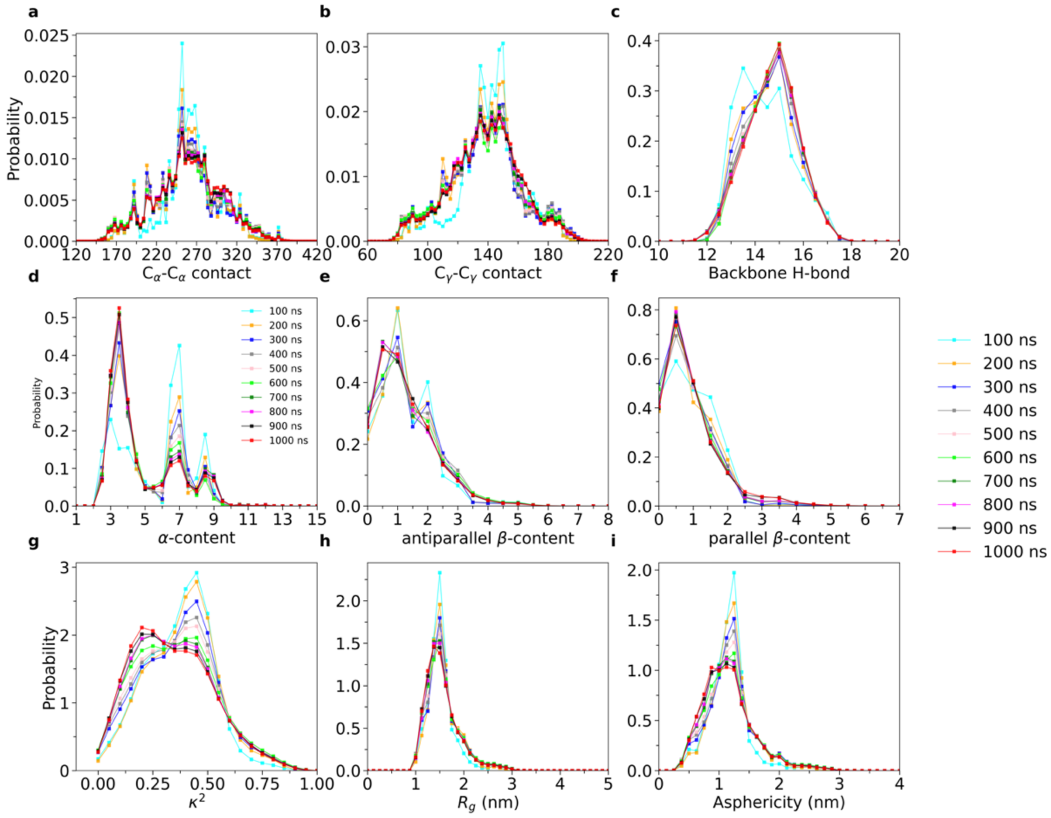

Figure S2 Time evolution of the one-dimensional probability densities of DHH1N as a function of single collective variables at 300 K for CHARMM36m PTMetaD-WTE.

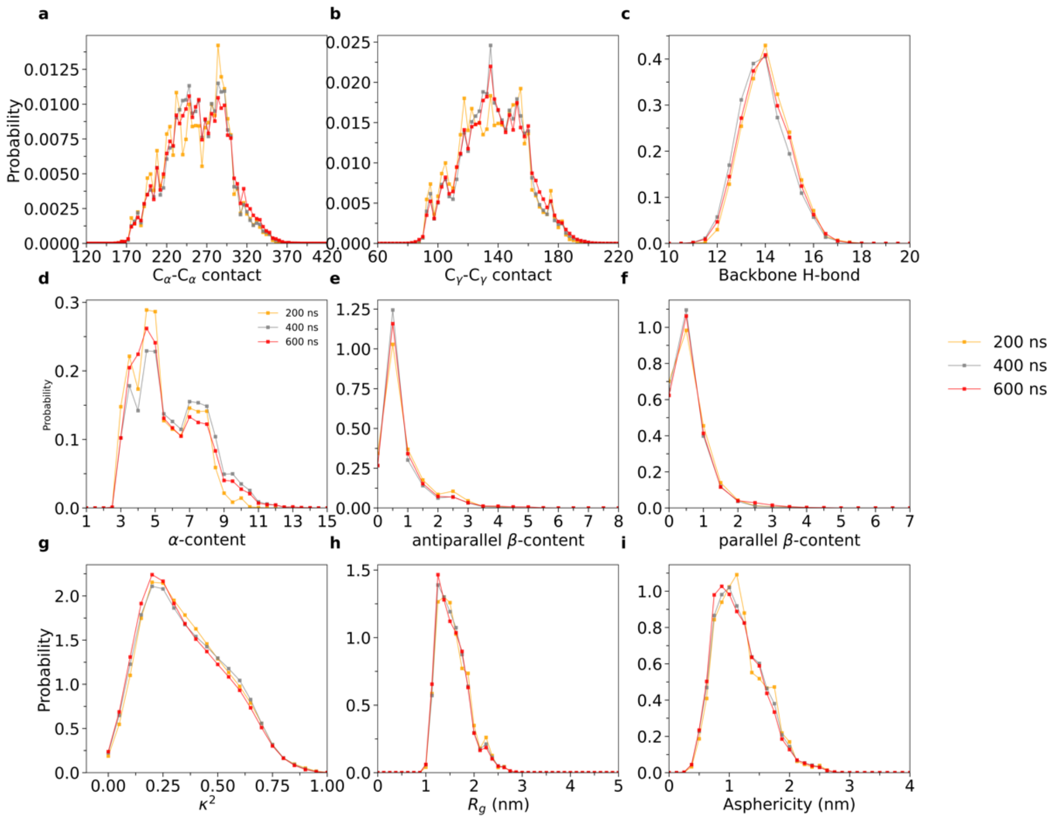

Figure S3 Time evolution of the one-dimensional probability densities of DHH1N as a function of single collective variables at 300 K for CHARMM22* PTMetaD-WTE.

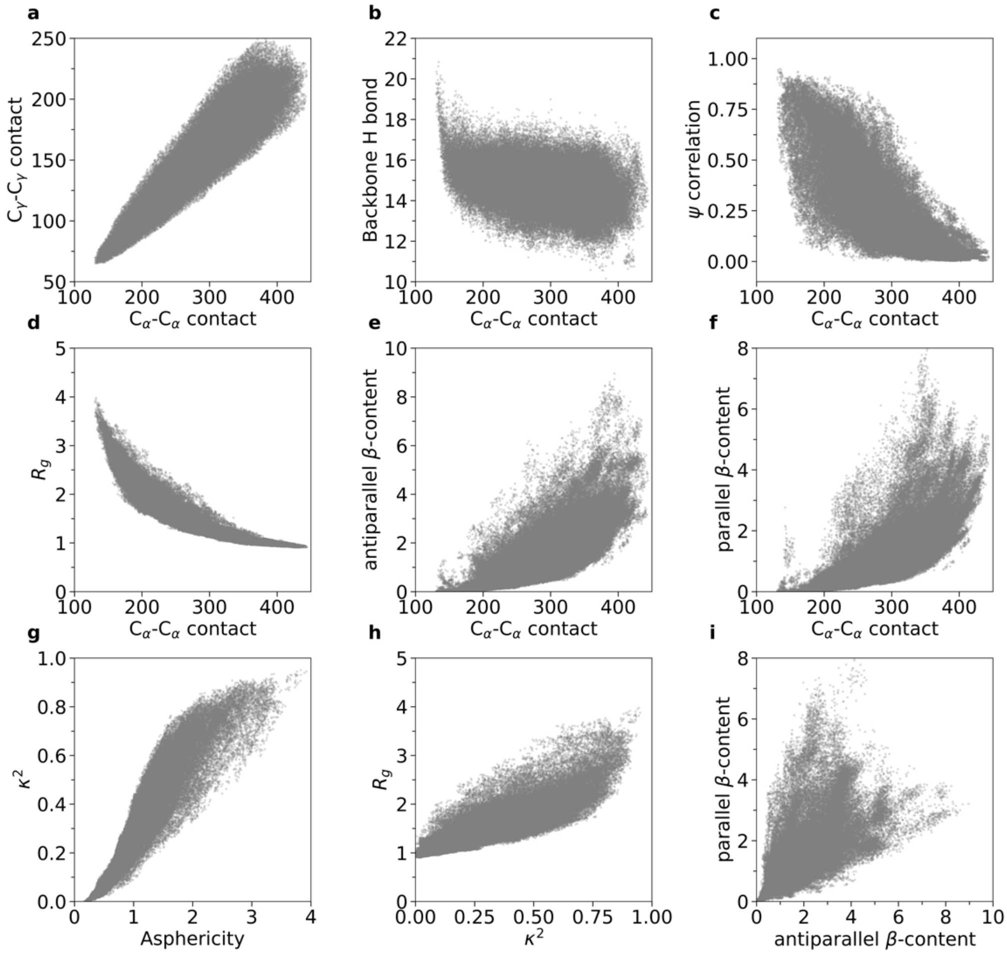

Figure S4 Correlation of the collective variables for DHH1N. Data are taken from the 300 K replica of CHARMM36m PTMetaD-WTE.

Supplementary Note 4

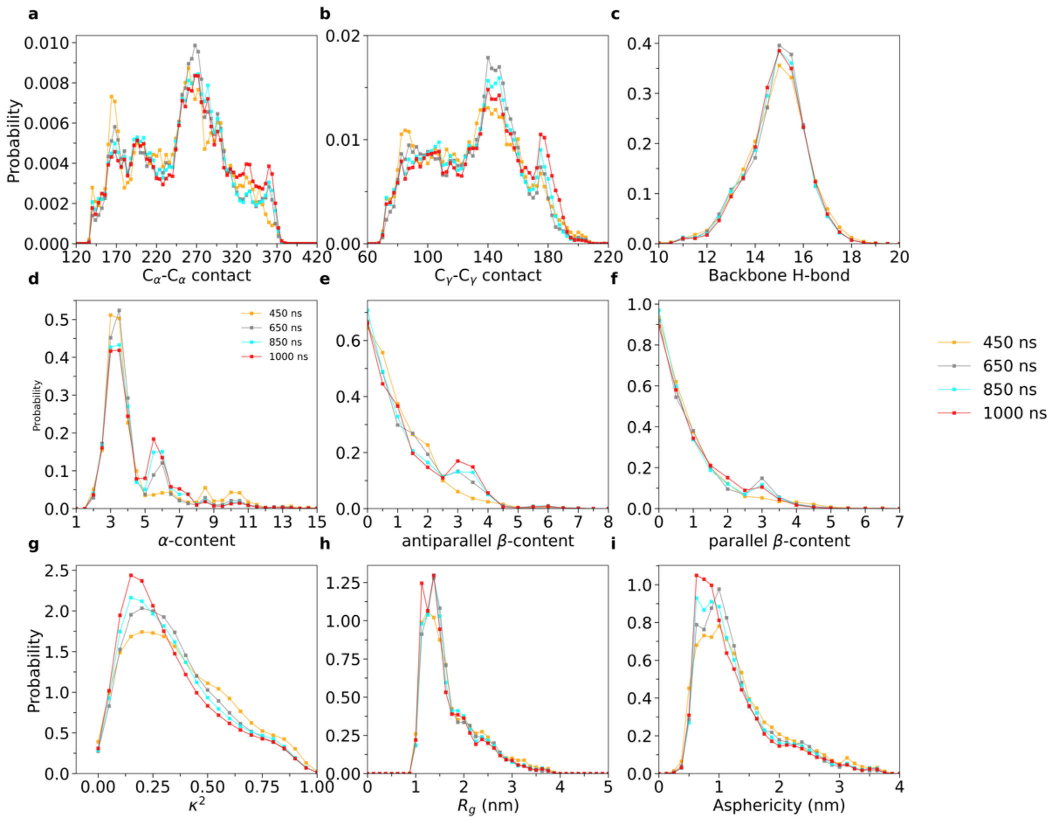

Figure S5 Time evolution of the one-dimensional probability densities of DHH1N as a function of single collective variables at 300 K for CHARMM36m BEMD. The first 250 ns was not included for data analysis.

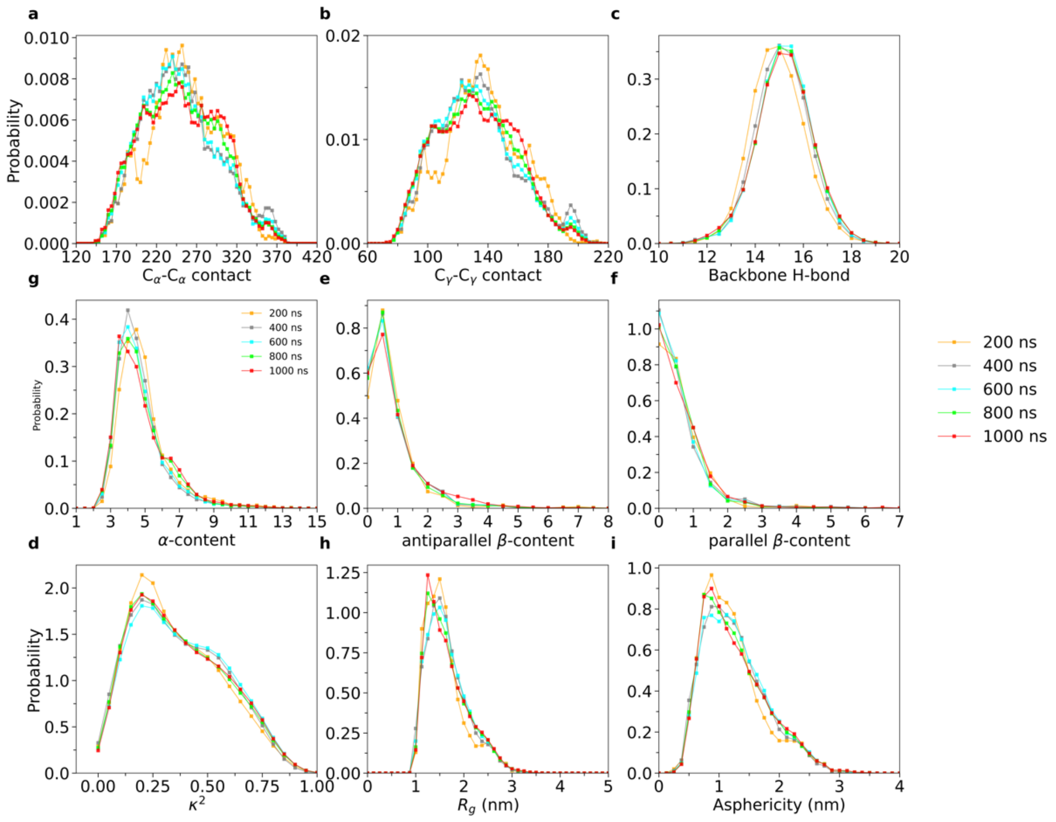

Figure S6 Time evolution of the one-dimensional probability densities of DHH1N as a function of single collective variables at 300 K for CHARMM22* BEMD.

Supplementary Note 5

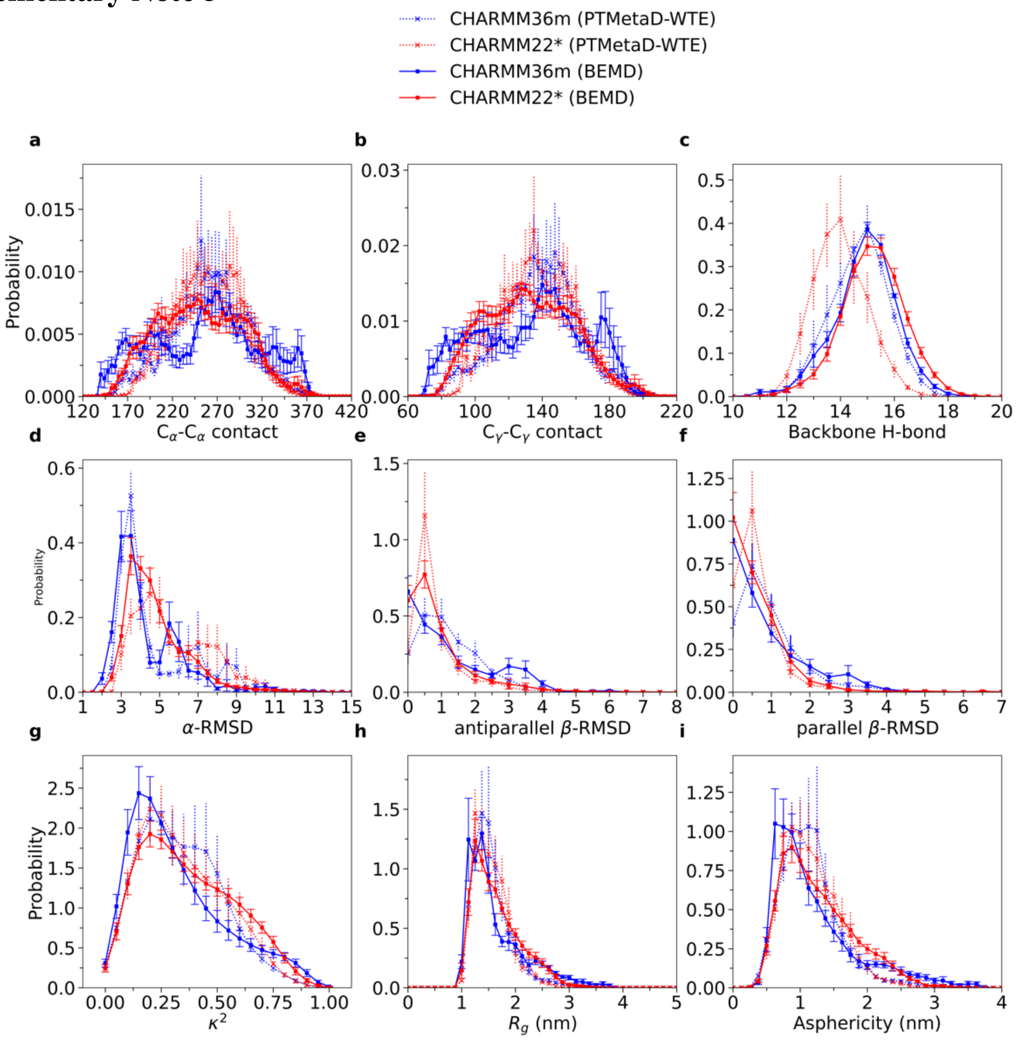

Figure S7 One-dimensional probability densities of DHH1N as a function of single collective variables at 300 K for CHARMM36m PTMetaD-WTE, CHARMM22* PTMetaD-WTE, CHARMM22* unbiased, CHARMM36m BEMD and CHARMM36m BEMD simulations.

Table S.3. Fraction of total equilibrium population (%) estimated from the histogram probability densities in Figure S7 d.

Cluster	PTMetaD-WTE		BEMD	
	CHARMM36m	CHARMM22*	CHARMM36m	CHARMM22*
1	70.2	51.8	71.9	70.1
2	21.3	36.6	23.8	26.2
3	8.5	11.6	4.3	3.7

Supplementary Note 6

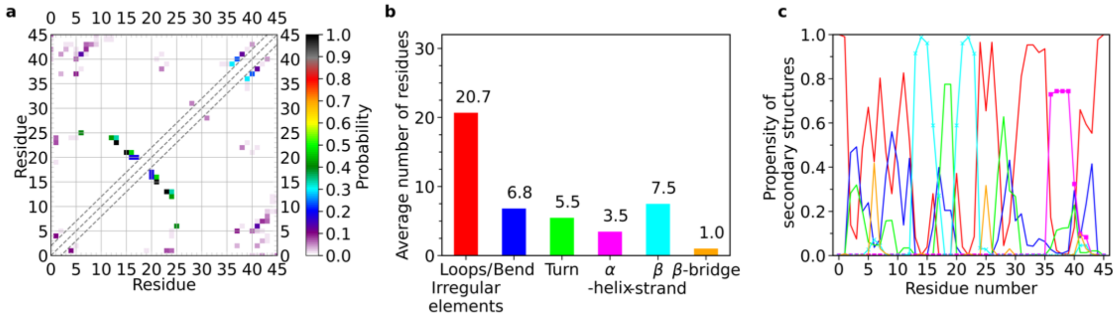

Figure S8 Contact-map and secondary-structure analysis for all conformations with concurring Asn14-Ala24 and Ser16-Lys22 contacts for CHARMM36m BEMD.

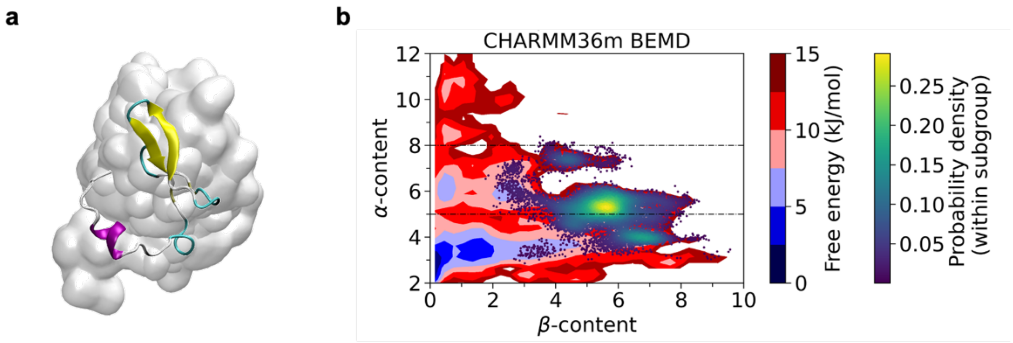

Figure S9 (a) Representative structure for the subgroup with concurring Asn14-Ala24 and Ser16-Lys22 contacts from CHARMM36m BEMD. The grey cloud represents the overlap of every 2000 subgroup trajectories; **(b)** 2D-FES on β-RMSD and α-RMSD for CHARMM36m BEMD and the scatter plot of Asn14-Ala24/ Ser16-Lys22 subgroup with probability densities relative to the total subgroup population.

Supplementary Note 7

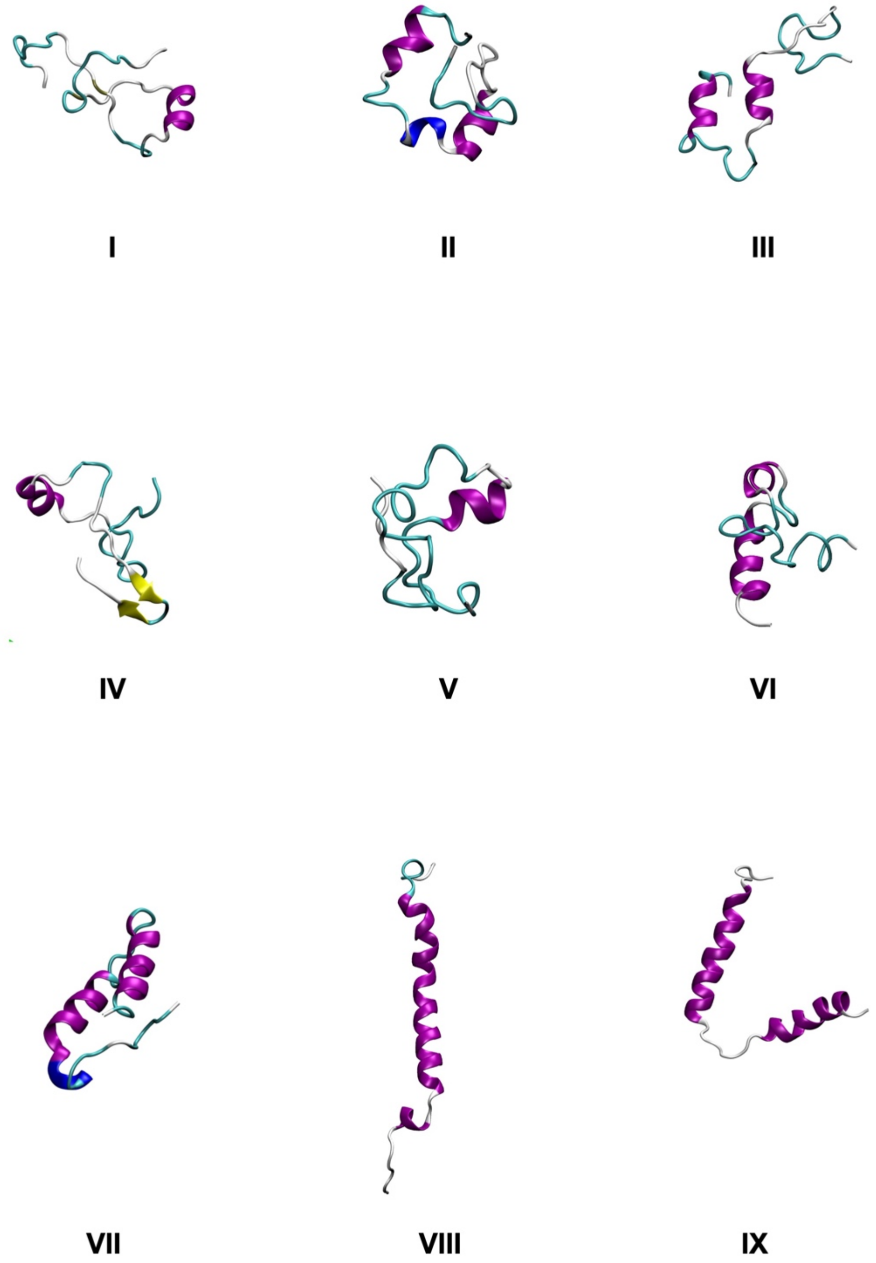

Figure S10 Nine initial structures for CHARMM22* unbiased simulations, built from i-Tasser[6] and Robetta[7].

Supplementary Note 8: input parameters for bias-exchange metadynamics simulations

Input parameter for the collective variables included in bias exchange metadynamics simulations are summarized in Table S.4 and Table S.5 for CHARMM22* and CHARMM36m force field, respectively. As described in the main text, simulations were performed adopting the ordinary metadynamics scheme; hills height is equal to 0.3 kJ mol^{-1} and bias potential is applied every 2500 simulation steps. After 160 ns the system explored a wide region for each collective variable and lower and upper boundaries were introduced as static harmonic bias potentials (LOWER_WALLS and UPPER_WALLS commands in PLUMED) to improve convergence.

Table S.4. Input parameters of BEMD and CHARMM22* force field. The parameter *k* is the harmonic constant for the static bias potential, and it is the same for lower and upper boundaries.

CV	σ	Lower wall	Upper wall	k [kJ CV^{-2} s^{-1}]
$C_\alpha - C_\alpha$ contacts	2.0	150	350	30
$C_\gamma - C_\gamma$ contacts	2.0	80	210	30
Backbone H-bonds	0.5	12	25	30
Dihedral correlation	1.0	10	40	30
Alpha helical content	0.1	2	15	30
Antiparallel beta sheet content	0.1	0	9	30
Parallel beta sheet content	0.1	0	8	30

Table S.5. Input parameters of BEMD and CHARMM36m force field. The parameter *k* is the harmonic constant for the static bias potential, and it is the same for lower and upper boundaries.

CV	σ	Lower wall	Upper wall	k [kJ CV^{-2} s^{-1}]
$C_\alpha - C_\alpha$ contacts	2.0	120	380	30
$C_\gamma - C_\gamma$ contacts	2.0	60	250	30
Backbone H-bonds	0.5	9	25	30
Dihedral correlation	1.0	5	40	30
α-helical content	0.1	1	20	30
Antiparallel β-sheet content	0.1	0	10	30
Parallel β-sheet content	0.1	0	12	30

Supplementary Note 9: summary of performed simulations.

Biased simulations – CHARMM22*

Table S.6. Summary of performed biased simulations with CHARMM22* force field. [a]Size of the system during *NVT* production phase after *NpT* equilibration. [b]Simulation time per replica.

**System**	**Box type**	**Box size [nm^3]**[a]	**Number of atoms**	**Water molecules**	**Simulation time [ns]**[b]
BEMD	Rhombic dodecahedron	398	40741	13344	1000
PTWTE	Rhombic dodecahedron	398	40741	13344	660

Biased simulations – CHARMM36m

Table S.7. Summary of performed biased simulations with CHARMM36m force field.

**System**	**Box type**	**Box size [nm^3]**[a]	**Number of atoms**	**Water molecules**	**Simulation time [ns]**[b]
BEMD	Rhombic dodecahedron	398	40741	13344	1000
PTWTE	Rhombic dodecahedron	494	49825	49825	1050

[a] Size of the system during *NVT* production phase after *NpT* equilibration.

[b] Simulation time per replica.

Unbiased simulations – CHARMM22*

Table S.8. Summary of performed unbiased simulations with CHARMM22* force field. [a]Structure numbering is the same of Figure S9. [b]Size of the system during *NVT* production phase after *NpT* equilibration.

**Input structure[a]**	**Box type**	**Box size [nm^3][b]**	**Number of atoms**	**Water molecules**	**Simulation time [ns]**
1	Cubic	240	24533	7948	1000
2	Cubic	205	20932	6741	1000
3	Cubic	400	40819	13370	1000
4	Cubic	242	24805	8032	1000
5	Cubic	170	17302	5531	1000
6	Cubic	156	16015	5102	1000
7	Cubic	170	17233	5508	1000
8	Cubic	352	35974	11755	1000
9	Cubic	690	70456	23249	1000
10	Cubic	310	31789	10360	1000

Supplementary Note 10: data analysis and error estimate

Postprocessing of PLUMED-defined collective variables was calculated using PLUMED 2.5.2[3]. 1D-FE profiles and 2D-FES were plotted with Matplotlib[8] and PyEMMA 2.5.7[9] in Jupyter Notebook[10]. Schematic visualisation of protein structures was produced using VMD[11] (http://www.ks. uiuc.edu/). Contact-map data were analysed using PyEMMA 2.5.7[9] and secondary-structure analysis was conducted using DSSP algorithm[12] in MDTraj 1.9.5[13] on Jupyter Notebook.

Error analysis for PTMetaD-WTE was conducted by means of Tiwary reweighting scheme[14] and block averages[15-17]. Error analysis for BEMD was conducted using block average[15-17] only because we analysed the unbiased replica. To estimate the statistical error for radius of gyration of CHARMM36m PTMetaD-WTE, we divided the trajectory into ten equal blocks with $N = 10$, each containing 10,000 frames (n_i = 10000) [100 ns of data per block for a total of 1000 ns of data]. The mean value $\bar{x}_i$ for each block was first calculated using the Tiwary reweighting scheme[14],

$$\bar{x}_i = \frac{\sum_{j=1}^{j=n_i} x_{i,j} w_{i,j}}{\sum_{j=1}^{j=n_i} w_{i,j}} \tag{S9}$$

with block number $i = 1, 2, \ldots, N$ till $N = 10$ and n_i the number of frames in each block.

The average $\bar{x}$ and its standard error were calculated:

$$\bar{x} = \frac{\sum_{i=1}^{i=N} \bar{x}_i}{N} \tag{S10}$$

$$\sigma^2 = \sqrt{\frac{\sum_{i=1}^{i=N}(x_i-\bar{x})^2}{(N-1)}} \qquad \text{(S11)}$$

We used a similar method to estimate the average and errors for secondary-structure analysis of CHARMM36m PTMetaD-WTE. DSSP assign a secondary-structure element to every residue for each frame. For every single frame, the total number of residues assigned to each secondary-structure element was summed up, and the mean total number of residues per element was calculated using the Tiwary reweighting scheme[14] for each of the ten block according to Eq. S9. The average number of residues belong to each secondary-structure element and its standard error were calculated using Eq. S10 and S11. Overall, we estimate the averages and standard errors for n_i = 250, 500, 1000, 2500, 5000, 7500, 10000, 15000 and 20000 respectively. Figure S10 below shows that $n_i \geq$ 2500 (25 ns of data per block) should give a good estimate of the standard error for the average number of residues assigned to Loops/irregular elements; Figure S11 below shows that $n_i \geq$ 10000 (100 ns of data per block) should give a good estimate of the standard error for the average number of residues assigned to α-helix. Overall, 100 ns of data per block was used throughout the work for all simulations.

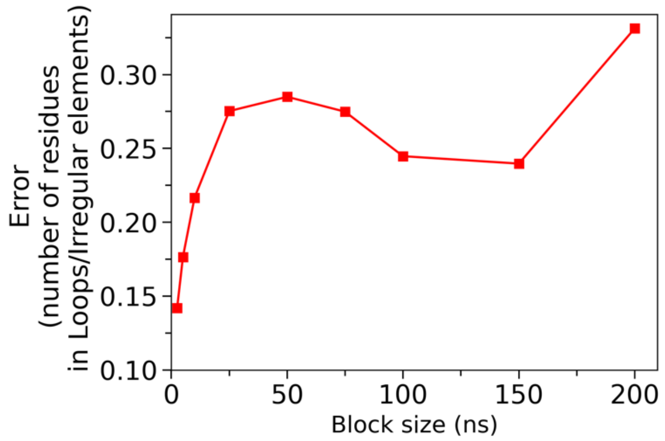

Figure S11 Evolution of the standard error on the average number of residues assigned to Loops/Irregular elements for CHARMM36m PTMetaD-WTE (Figure 3b) with the size of block.

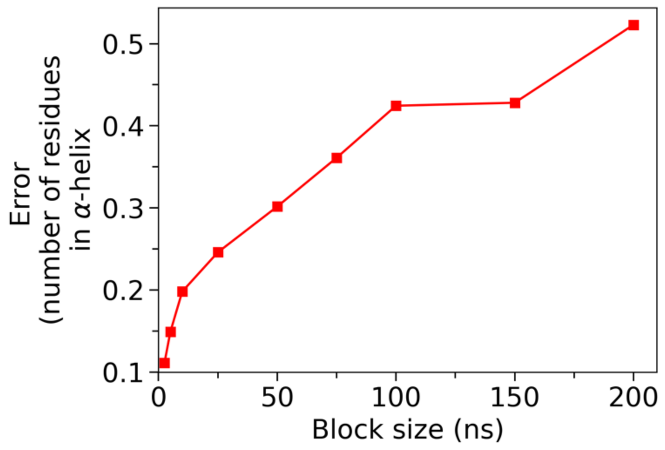

Figure S12 Evolution of standard error on the average number of residues assigned to α-helix for CHARMM36m PTMetaD-WTE (Figure 3b) with the size of block.

To estimate the statistical errors of the 1D-FE profiles and probability density distributions (e.g., Figure 1 and Figure S1), we used the same blocking approach as described above, with 100 ns of data per block and a total of 10 block in most cases. Within each block, a histogram was calculated using kernel density estimation via the HISTOGRAM command in PLUMED with proper Tiwary reweighting[17], after which the ten histograms were block-averaged and normalized to obtain a mean probability density distribution $P(s)$ with associated error bars (based on the python code in https://www.plumed.org/doc-v2.6/user-doc/html/lugano-4.html). With s representing each of the PLUMED-defined collective variables, the 1D-FE profiles $F(s)$ was then obtained by inverting the probability density distribution according to:

$$F(s) = -k_B T \ln P(s) \quad \text{(S10)}$$

To calculate the combined errors for the unbiased CHARMM22*, block analysis for each of the nine replicas was conducted, after which the final error is calculated from individual errors by the following Equation,

$$\sigma = \frac{\sqrt{\sum_{i=1}^{i=9} \sigma_i^2}}{9} \tag{S11}$$

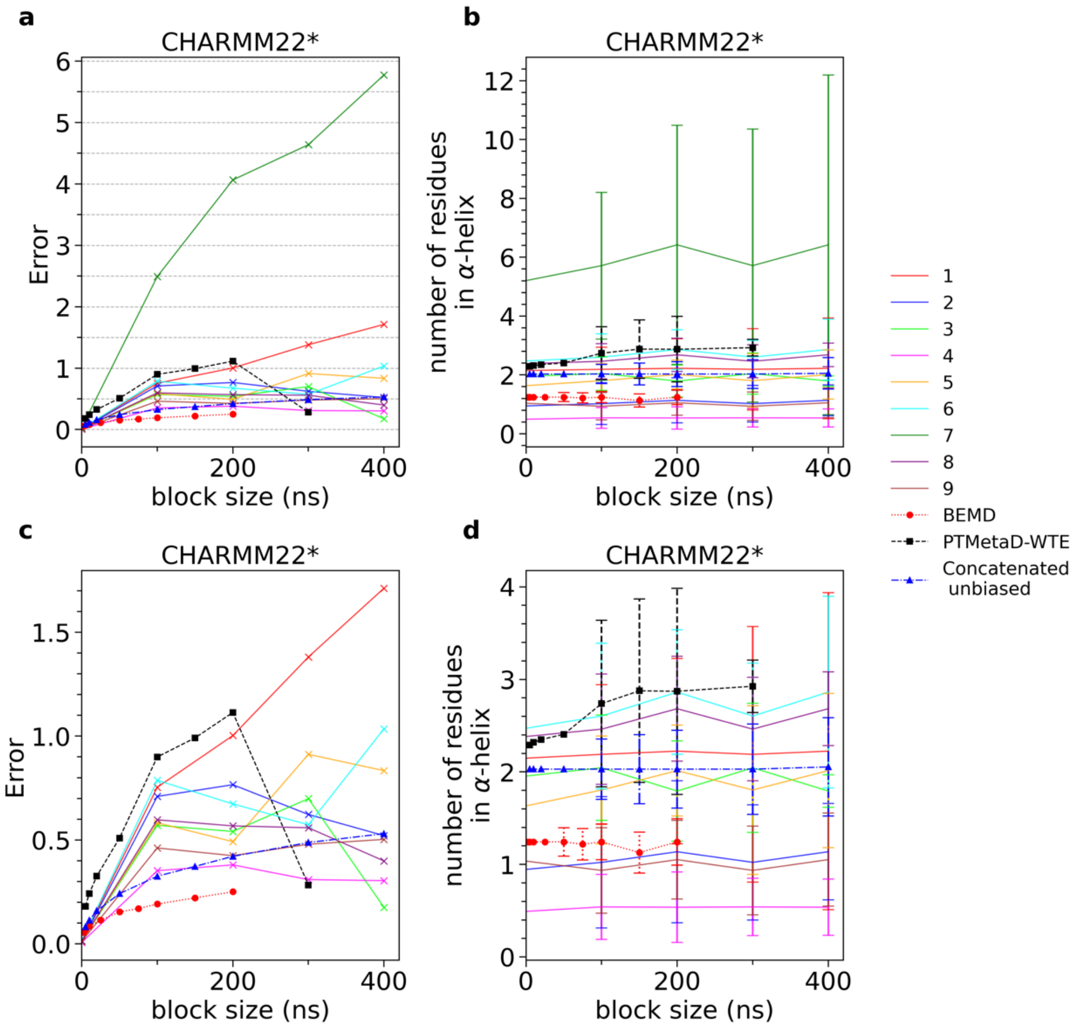

Figure S13. Error analysis of α-helical residues for unbiased CHARMM22*, CHARMM22* BEMD and CHARMM22* PTMetaD-WTE. The numbers refer to the initial structures of the nine independent unbiased simulations, and the concatenated unbiased refer to concatenating the nine data sets into a single trajectory for analysis (as in the original manuscript). **c)** and **d)** have excluded the data of structure 7.

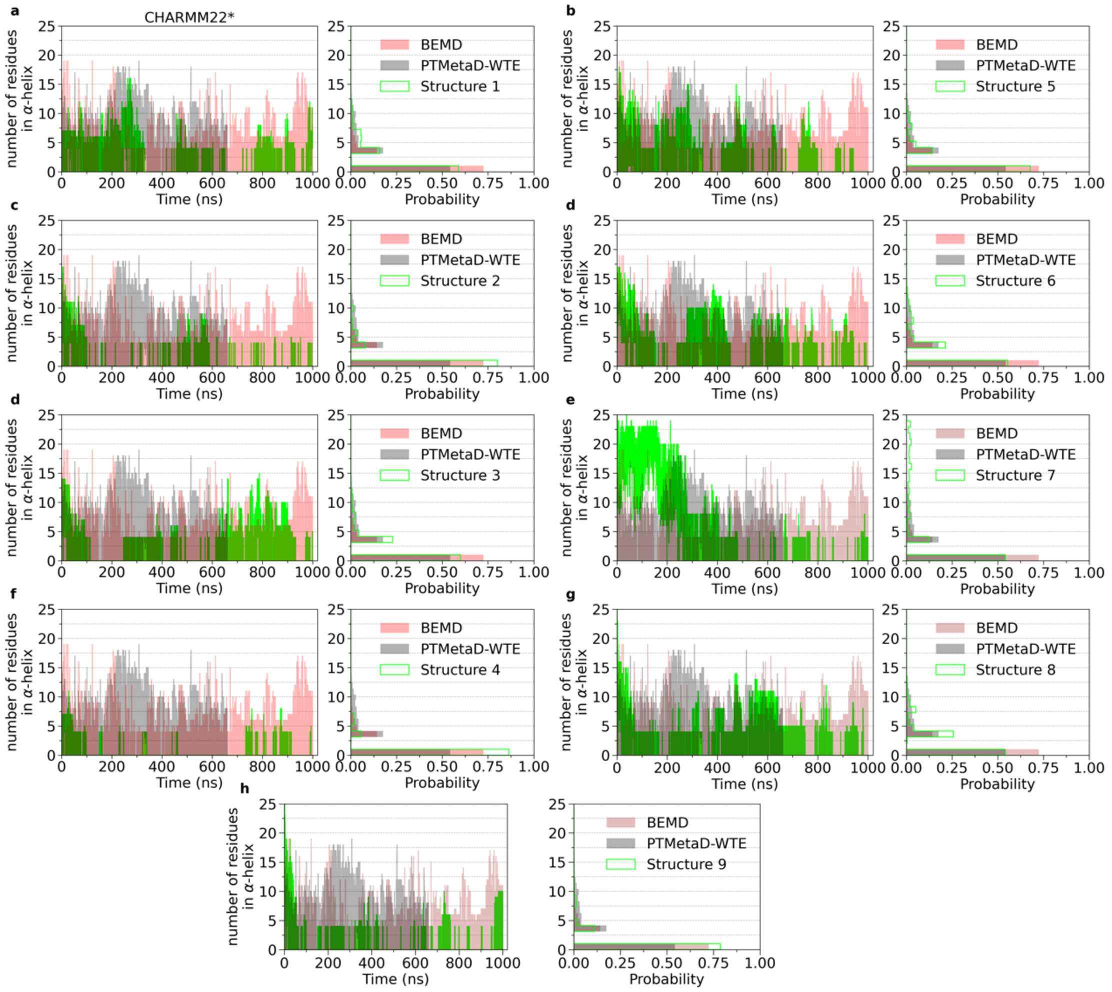

Figure S14. Time evolution and probability distribution ofα-helical residues for unbiased CHARMM22*, CHARMM22* BEMD and CHARMM22* PTMetaD-WTE. The structure numbers refer to the initial structures of the nine independent unbiased simulations.

REFERENCES

1. Hondele, M., Sachdev, R., Heinrich, S., Wang, J., Vallotton, P., Fontoura, B. M. and Weis, K. DEAD-box ATPases are Global Regulators of Phase-separated Organelles. *Nature* **2019**, *573(7772)*, 144-148.
2. Linsenmeier, M., Kopp, M. R., Grigolato, F., Emmanoulidis, L., Liu, D., Zürcher, D., Hondele, M., Weis, K., Capasso Palmiero, U. and Arosio, P. Dynamics of Synthetic Membraneless Organelles in Microfluidic Droplets. *Angew. Chem.* **2019**, *131(41)*, 14631-14636.
3. Tribello, G. A., Bonomi, M., Branduardi, D., Camilloni, C. and Bussi, G. PLUMED 2: New Feathers for An Old Bird. *Comput. Phys. Commun.* **2014**, *185(2)*, 604-613.
4. Pietrucci, F. and Laio, A. A Collective Variable for the Efficient Exploration of Protein Beta-Sheet Structures: Application to SH3 and GB1. *J. Chem. Theory Comput.* **2009**, *5(9)*, 2197-2201.
5. Vymětal, J. and Vondrášek, J. Gyration-and Inertia-tensor-based Collective Coordinates for Metadynamics. Application on the Conformational Behavior of Polyalanine Peptides and Trp-cage folding. *J. Phys. Chem. A.* **2011**, *115(41)*, 11455-11465.
6. Yang, J. and Zhang, Y. "I-TASSER Server: New Development for Protein Structure and Function Predictions." *Nucleic Acids Res.* **2015**, *43(W1)*, W174-W181.
7. Song, Y., DiMaio, F., Wang, R. Y. R., Kim, D., Miles, C., Brunette, T. J., Thompson, J. and Baker, D. High-Resolution Comparative Modeling with Rosettacm. *Structure* **2013**, *21(10)*, 1735-1742.
8. Hunter, John D. Matplotlib: A 2D Graphics Environment. *Comput. Sci. Eng.* **2007**, *9(03)*, 90-95.
9. Scherer, M.K., Trendelkamp-Schroer, B., Paul, F., Pérez-Hernández, G., Hoffmann, M., Plattner, N., Wehmeyer, C., Prinz, J.H. and Noé, F. PyEMMA 2: A Software Package for

Estimation, Validation, And Analysis of Markov Models. *J. Chem. Theory Comput.* **2015**, 11(11), 5525-5542.

10. Kluyver, T., Ragan-Kelley, B., Pérez, F., Granger, B.E., Bussonnier, M., Frederic, J., Kelley, K., Hamrick, J. B., Grout, J., Corlay, S., Ivanov, P., Avila, D., Abdalla, S., Willing, C. and Jupyter Development Team. Jupyter Notebooks—A Publishing Format for Reproducible Computational Workflows. In Positioning and Power in Academic Publishing: Players, Agents and Agendas (eds Loizides, F. & Schmidt, B.) 87-90 (IOS Press, 2016).
11. Humphrey, W., Dalke, A. and Schulten, K. VMD: Visual Molecular Dynamics. Journal Of Molecular Graphics *J. Mol. Graph.* **1996**, *14(1)*, 33-38.
12. Kabsch, W. and Sander, C. Dictionary of Protein Secondary Structure: Pattern Recognition of Hydrogen-Bonded and Geometrical Features. *Biopolymers* **1983**, *22(12)*, 2577-2637.
13. McGibbon, R.T., Beauchamp, K.A., Harrigan, M.P., Klein, C., Swails, J.M., Hernández, C.X., Schwantes, C.R., Wang, L.P., Lane, T.J. and Pande, V.S. MDTraj: A Modern Open Library for The Analysis of Molecular Dynamics Trajectories. *Biophys. J.* **2015**, 109(8), 1528-1532.
14. Tiwary, P. and Parrinello, M. A Time-independent Free Energy Estimator for Metadynamics. *J. Phys. Chem. B* **2015**,119(3), 736-742.
15. Frenkel, D. and Smit, B. *Understanding Molecular Simulation: From Algorithms to Applications*; Elsevier; 2001.
16. Flyvbjerg, H. and Petersen, H.G. Error Estimates on Averages of Correlated Data. *J. Chem. Phys.* **1989**, *91(1)*, 461-466.

17. Bussi, G. and Tribello, G.A. Analyzing and Biasing Simulations with PLUMED. *Biomolecular Simulations*; Methods in Molecular Biology; Humana Press, 2019; Vol. 2022, pp 529– 578.
